# SARS‐CoV‐2 triggered oxidative stress and abnormal energy metabolism in gut microbiota

**DOI:** 10.1002/mco2.112

**Published:** 2022-01-17

**Authors:** Tuoyu Zhou, Jingyuan Wu, Yufei Zeng, Junfeng Li, Jun Yan, Wenbo Meng, Huawen Han, Fengya Feng, Jufang He, Shuai Zhao, Ping Zhou, Ying Wu, Yanlin Yang, Rong Han, Weilin Jin, Xun Li, Yunfeng Yang, Xiangkai Li

**Affiliations:** ^1^ Ministry of Education Key Laboratory of Cell Activities and Stress Adaptations School of Life Sciences Lanzhou University Lanzhou P. R. China; ^2^ Gansu Province Key Laboratory Biotherapy and Regenerative Medicine The First Hospital of Lanzhou University Lanzhou P. R. China; ^3^ State Key Joint Laboratory of Environment Simulation and Pollution Control School of Environment Tsinghua University Beijing P. R. China; ^4^ Medical Frontier Innovation Research Center The First Hospital of Lanzhou University Lanzhou P. R. China

**Keywords:** COVID‐19, gut microbiota, metagenome, metatranscriptome, SARS‐CoV‐2

## Abstract

Specific roles of gut microbes in COVID‐19 progression are critical. However, the circumstantial mechanism remains elusive. In this study, shotgun metagenomic or metatranscriptomic sequencing was performed on fecal samples collected from 13 COVID‐19 patients and controls. We analyzed the structure of gut microbiota, identified the characteristic bacteria, and selected biomarkers. Further, gene ontology (GO) and Kyoto Encyclopedia of Genes and Genomes (KEGG) annotations were employed to correlate the taxon alterations and corresponding functions. The gut microbiota of COVID‐19 patients was characterized by the enrichment of opportunistic pathogens and depletion of commensals. The abundance of *Bacteroides* spp. displayed an inverse relationship with COVID‐19 severity, whereas *Actinomyces oris*, *Escherichia coli*, and *Streptococcus parasanguini* were positively correlated with disease severity. The genes encoding oxidoreductase were significantly enriched in gut microbiome of COVID‐19 group. KEGG annotation indicated that the expression of ABC transporter was upregulated, while the synthesis pathway of butyrate was aberrantly reduced. Furthermore, increased metabolism of lipopolysaccharide, polyketide sugar, sphingolipids, and neutral amino acids were found. These results suggested the gut microbiome of COVID‐19 patients was in a state of oxidative stress. Healthy gut microbiota may enhance antiviral defenses via butyrate metabolism, whereas the accumulation of opportunistic and inflammatory bacteria may exacerbate COVID‐19 progression.

## Graphical abstract

The gut microbiota of COVID‐19 patients showed upregulation of oxidative stress and inflammation response, accompanied by depletion of symbiotic bacteria. The abundance of *Bacteroides* spp. is negatively correlated with disease severity, while the accumulation of opportunistic pathogens may exacerbate the progression of COVID‐19. Healthy intestinal flora may enhance the organic defense against SARS‐CoV‐2 virus infection through butyrate metabolism.

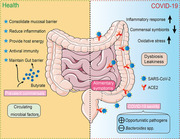

## 1 INTRODUCTION

The COVID‐19 pandemic caused by severe acute respiratory syndrome coronavirus 2 (SARS‐CoV‐2) triggered acute and severe respiratory pathology, and growing evidence suggested that complicating gastrointestinal symptoms is common as extrapulmonary manifestations.^1^, ^2^, ^3^ Virus ribonucleic acid (RNA) was detected both in fecal and anal swab of SARS‐CoV‐2 infected patients,^4^ while high load of anal swab virus had been associated with adverse clinical outcomes in patients. In addition, SARS‐CoV‐2 had also been found to coexist with oral microorganisms in oral environment.^5^, ^6^ Further, some cases suggested that untreated sewage might increase the fecal‐oral transmission risk of the virus.^7^, ^8^ SARS‐CoV‐2 infects host cells through the ACE2 receptor^9^ and continuously replicates in the gastrointestinal system,^10^ thereby weakening the intestinal barrier. It had been authenticated that ACE2 was a vital regulator of intestinal inflammation,^11^ and the deficiency of which may alter the inflammatory sensitivity, thus aggravating the gut microbiota imbalance and gastroenteritis‐like symptoms.^12^

Gut microbiota provides various biological functions for the host, including promoting immune system homeostasis, metabolizing nutrients, and maintaining the intestinal mucosal barrier.^13^ At the same time, gut microbiota is also thought to be a contributing factor in virus clearance.^14^, ^15^ In contrast, the gut microbiota dysbiosis reduces antiviral immune responses and aggravated respiratory diseases.^16^ Consumption of antibiotic‐sensitive gut microbes could augment the susceptibility to pulmonary allergic inflammation and influenza virus infection.^17^ Severe influenza A virus infection was associated with intestinal disease and altered gut microbiota.^18^ The greater abundance of *Escherichia coli* and *Enterococcus faecium* in the H7N9 patients might be account for bacteremia and abdominal infection.^19^

Existing studies described the close link between microbiota dysbiosis and SARS‐CoV‐2 infection.^5^, ^6^, ^20^ Compared with healthy controls, COVID‐19 patients showed significantly lower bacterial diversity,^21^ while opportunistic pathogens enrichment and beneficial bacteria depletion were also observed.^21^, ^22^ Some of the reduced symbiotic bacteria were from the *Ruminococcaceae* and *Lachnospiraceae* families, including *Ruminococcaceae* UCG_013, *Ruminococcus obeum*, *Ruminococcus bromii*, and *Anaerostipes*, *Agathobacter*, *Dorea formicigenerans*, *Fusicatenibacter roseburia*, respectively.^22^ Besides, the butyrate‐producing bacterium *Faecalibacterium prausnitzii* was found to be negatively associated with COVID‐19 severity.^23^, ^24^ In contrast, two pathogenic *Clostridiums* (*C. ramosum* and *C. hathewayi*) were correlated to the disease severity.^22^ Notably, some specific *Bacteroides* spp., capable of down‐regulating ACE2 expression in the murine gut, are inversely correlated with the SARS‐CoV‐2 load.^22^ These results highlight the potential role of gut microbiota in the disease predisposition of COVID‐19 patients. Nevertheless, the specific mechanism of interaction between SARS‐CoV‐2 and gut microbiota remains elusive. Especially, the association between taxon and related functions should be explored in depth.

Taken together, SARS‐CoV‐2 invasion of intestinal epithelial cells and block of ACE2 receptor may alter cell metabolic status,^25^, ^26^, ^27^ damage intestinal barriers, and form specific immune inflammatory environment in gastrointestinal tract,^28^, ^29^ thus changing the composition and function of intestinal microorganisms in COVID‐19 patients. On the other hand, the presence of symbiotic microorganisms determines host immunity, while the composition of gut microbiota could influence the susceptibility to SARS‐CoV‐2.^30^ Thus, we hypothesized that the gut microbiota of COVID‐19 patients is significantly different from that of healthy people, specific bacterial species may critically maintain immune homeostasis and energy supply against COVID‐19 development.^31^ Through metagenome (MG) and metatranscriptome (MT) sequencing, this study annotated the gut microbiome information of COVID‐19 patients and described the alterations in core microbial communities with their related functions. Additionally, we revealed the connection between these changes and clinic features of COVID‐19 patients, further elucidating their interactions in active transcripts features.

## 2 RESULTS

### 2.1 Information of subjects

**FIGURE 1:**
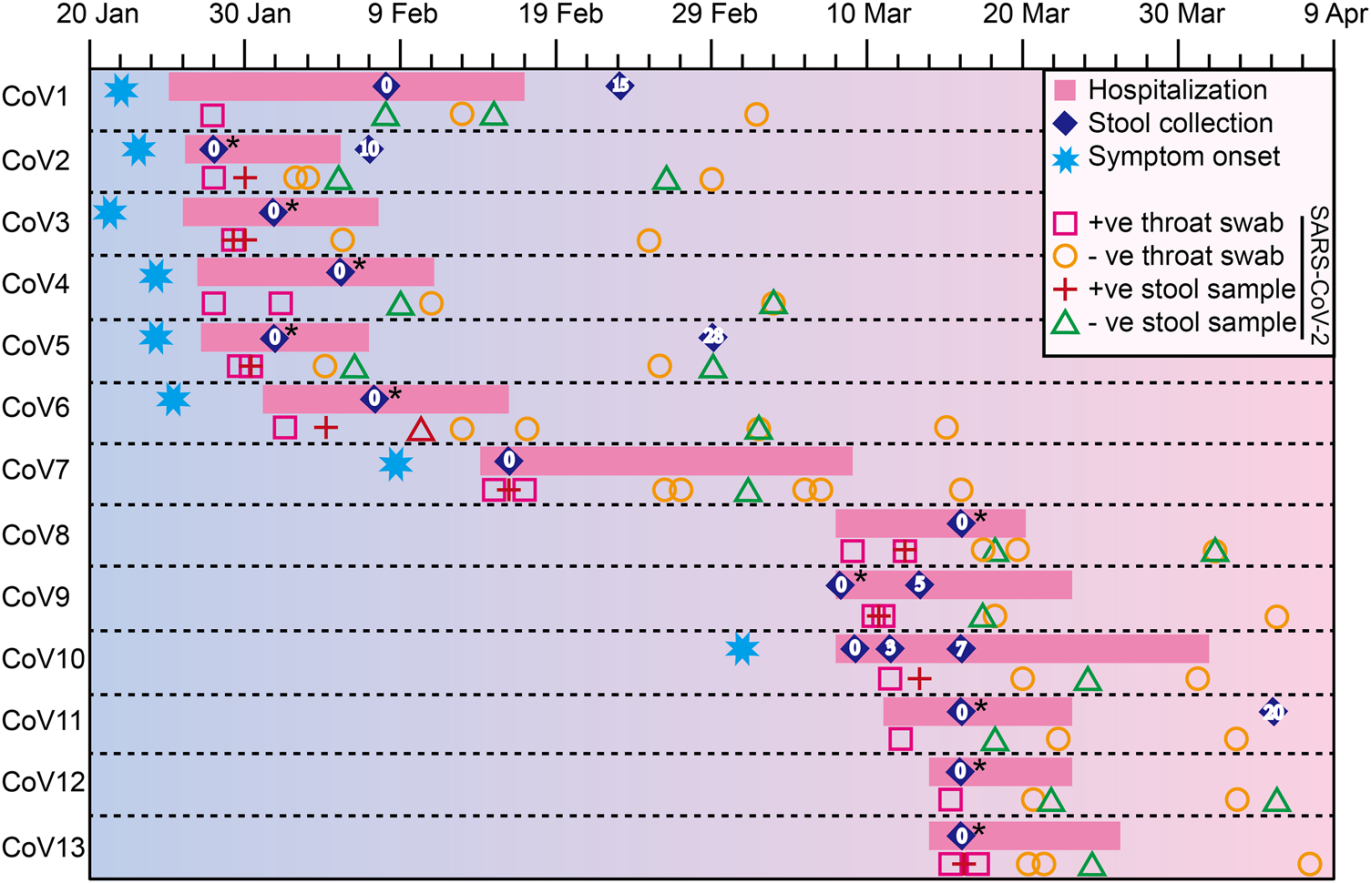
Schematic diagram of fecal specimen collection in COVID‐19 patients. “CoV” indicates COVID‐19 patients. “0” represents the baseline date of the first feces collection; “+ve stool sample”: the positive qRT‐PCR test result for SARS‐CoV‐2 in stool specimen; “−ve stool sample”: the negative qRT‐PCR test result for SARS‐CoV‐2 in stool specimen. “+ve throat swab”: the positive qRT‐PCR test result for SARS‐CoV‐2 in throat swab; “−ve throat swab”: the negative qRT‐PCR test result for SARS‐CoV‐2 in throat swab test. Fecal specimens sequenced by both shotgun metagenome and metatranscriptome sequencing were marked with asterisk symbols

All COVID‐19 patients (13 cases) enrolled in this study were cured and discharged from hospital. Before discharge, the quantitative real‐time polymerase chain reaction (qRT‐PCR) test results for SARS‐CoV‐2 in throat swab and stool specimens of all COVID‐19 patients were negative. Follow‐up survey after discharge indicated that SARS‐CoV‐2 virus was still negative in throat swab or stool specimens of all subjects (Figure 1). The 13 COVID‐19 patients included one severe case, four moderate cases, and eight mild cases (Table S2). Sixty‐two percent COVID‐19 patients received empirical antibiotics treatment, while five of them were not exposed to antibiotics. All COVID‐19 patients received antiviral therapy, and 12 of them were treated with interferon α and Kaletra (Table 1 and Table S2). The age range of COVID‐19 patients is from 21 to 50 years old, with a median age of 24, and the majority are male, accounting for approximately 76% (Table 1). Among them, eight COVID‐19 cases developed clinical symptoms including fever, cough, sore throat, and chest distress (Table 1), and five cases were asymptomatic carriers. However, only one patient was reported gastrointestinal discomfort symptoms during hospitalization. Computed tomography (CT) scans also showed 38% of COVID‐19 patients developed ground‐glass lung appearance. The results of biochemical indicators elicited that the lymphocyte and activated partial thromboplastin time (APTT) levels of COVID‐19 patients were significantly lower than those in health cohort, while hemoglobin (HGB) and total bilirubin (TIBIL) levels were significantly higher than those in health group (Figure S2, Table S1). Although most physiological indicators of community‐acquired pneumonia group (CAP) and COVID‐19 patients had similar trends (Figure S2, Table S3), the levels of globulin, D‐dimer, and fibrinogen in CAP‐group were significantly higher than that in health controls, while these parameters in COVID‐19 patients were much closer to that of healthy controls (Figure S2, Table S3).

**TABLE 1:** Characteristics of all subjects

Variables	COVID‐19 cases	CAP patients	Health controls
Numbers	13	24	13
Median Age, years (IQR)	24 (22.5, 45.5)	32 (29, 40)	26 (23, 45.5)
Male	10 (76%)	12 (50%)	10 (76%)
Signs and symptoms at admission			
Fever	5 (38%)	23 (96%)	NA
Cough	5 (38%)	15 (62%)	
Sore throat	3 (23%)	5 (20%)	
Chest distress	1 (8%)	0 (0%)	
Diarrhea	1 (8%)	0 (0%)	
Chest computed tomography scan			
Lung markings increased	6 (46%)	5 (20%)	NA
Mottling and ground‐glass opacity	5 (38%)	20 (83%)	
Antibiotic therapy at presentation			
Ceftriaxone	2 (15%)	4 (17%)	NA
Moxifloxacin	7 (53%)	19 (80%)	
Levofloxacin	1 (7%)	2 (8%)	
Antiviral therapy			
Oseltamivir	2 (15%)	5 (21%)	NA
Interferon alpha	12 (92%)	4 (17%)	
Kaletra	12 (92%)	0 (0%)	
Ribavirin	1 (8%)	5 (21%)	
Death	0 (0%)	0 (0%)	NA

*Note*: Values are expressed in number (percentage) and median (interquartile range).

Abbreviations: CAP, community‐acquired pneumonia; NA, not available.

### 2.2 Gut microbiota structure dissimilarity among COVID‐19, health, and CAP groups

**FIGURE 2:**
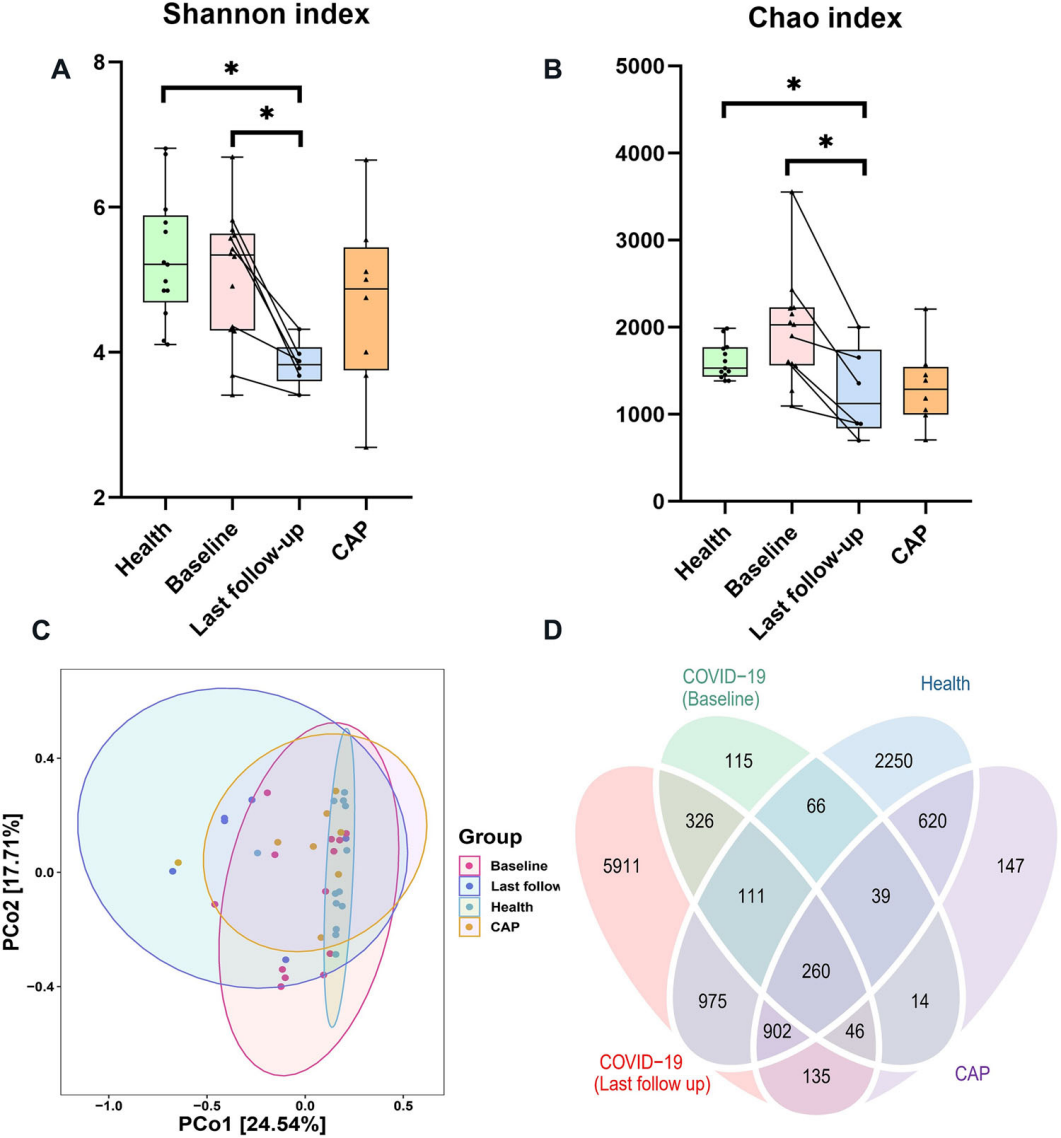
Alteration in gut microbial diversity and community structures in COVID‐19 (*n* = 13), health (*n* = 13) and community‐acquired pneumonia (CAP) (*n* = 8) groups. Alpha diversity of the gut microbiota among the three groups based on the (A) Shannon index and (B) Chao index. (C) Microbiome communities were assessed by principal coordinate analysis (PCoA) of Bray–Curtis distances. (D) Venn diagram presenting the overlap of operational taxonomic units (OTUs) of the fecal microbiome across all groups. Significance was marked as *p < 0.05, **p < 0.01, ***p < 0.001

MG sequencing was performed on fecal samples from the COVID‐19/Health/CAP groups, resulting in raw reads/clean reads of 11.98/10.88, 11.24/10.43, and 13.33/10.11 giga, respectively (Table S4). Then, we evaluated the impact of clinical management and individual differences on intestinal microbiota by PERMANOVA. COVID‐19 had the greatest impact on fecal microbiota (PERMANOVA test, *R* ^2 ^= 0.06, *p* = 0.02), while age, sex, antibiotics, and antiviral drugs had no significant impact (Figure S3). Next, the samples of COVID‐19 patients were divided into baseline and last follow‐up groups. Albeit the Shannon index and Chao index of the baseline samples in the COVID‐19 group were close to those in the health group, these indexes based on the last follow‐up samples were significantly decreased (Figure 2A,B). PCoA plot indicated the fecal microbiome of healthy subjects clumped together, while the samples of COVID‐19 group developed stronger heterogeneity (Figure 2C and Figure S4). In addition, PERMANOVA and ANOSIM analysis also showed significant differences in the intestinal microbiota structure between COVID‐19 patients and healthy volunteers (Table S6). Microbes present in all samples from each subject group were defined as core microbes. At the level of species, the number of core bacteria in COVID‐19 (baseline, last follow‐up) was respectively 716 and 626, and the number in CAP and Health groups was 609 and 837, respectively. Subsequently, all levels of core microorganisms were represented by venn diagram. All three groups shared 1201 core microbes, while COVID‐19 and health groups owned 2241 common microbes (Figure 2D). Notably, there were significant differences in core microorganisms between baseline and last follow‐up samples, with an overlap rate of only 8%. In addition, the mean Bray–Curtis distances in Baseline were remarkably higher than that of health group (Kruskal–Wallis test, *p* < 0.001, Figure S5).

### 2.3 Taxa composition of COVID‐19, health, and CAP groups

**FIGURE 3:**
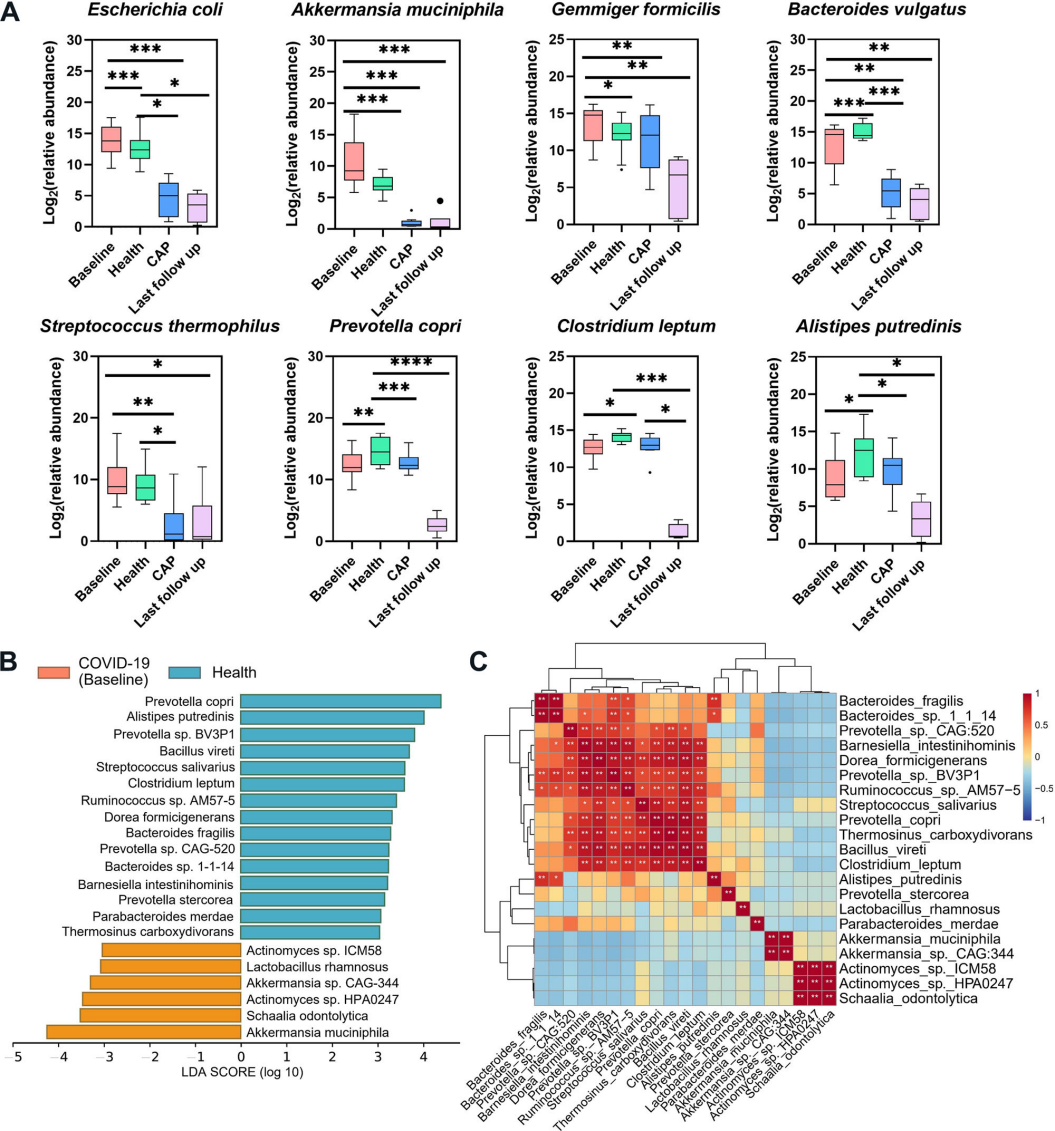
Taxonomic differences in the stool microbiota between COVID‐19 and control groups. (A) Comparison of the relative abundance at the species levels across all groups. Specific to box figure, each box corresponds to an interquartile range of taxa abundance, and the black line represents to median abundance. Vertical lines indicate the variability in the abundance of each taxon. Significance was marked as *p < 0.05, **p < 0.01, ***p < 0.001. (B) LEfSe analysis conducted to reveal the significant differences in microbiota composition between COVID‐19 (orange) and health (blue) groups. (C) Pearson correlation of associated species in COVID‐19 and health groups. The degree of correlation is indicated by a color gradient from red (positive correlation) to blue (negative correlation)

To investigate the alteration of microbiota taxa, the relative proportion of microorganisms was assessed at the species levels (Figure 3A, Tables S7 and S8). Compared with health group, the abundance of *Bacteroides vulgatus*, *Prevotella copri, Clostridium leptum*, and *Alistipes putredinis* was decreased in COVID‐19 (Baseline) and CAP groups, while the abundance of *E. coli*, *Akkermansia muciniphila*, and *Gemmiger formicili* was increased only in baseline samples. It should be noted that the relative abundance of *Streptococcus thermophil* exhibited no statistical difference in health group compared with baseline, but significantly higher in contrast to CAP group. In addition, there were still differences in bacterial abundance between last follow‐up and health/baseline groups. The result of linear discriminant analysis effect size (LEfSe) anlysis identified that *Actinomyces sp*. ICM58, *Actinomyces sp*. HPA0247*, Schaalia odontolytica, A. muciniphila, Akkermansia sp*. CAG_344, and *Lactobacillus rhamnosus* dominated baseline group (Figure 3B). Besides, last follow‐up group was characterized with *Klebsiella pneumoniae*, *E. coli*, *Shigella dysenteriae*, and *Shigella flexneri* (Table S9). Pearson correlation analysis revealed a significant negative correlation between the characteristic bacteria in COVID‐19 and health groups (Figure 3C). The 30 most dominant species based on the random forest model (Figure S6A,B) were compared with the LEfSe results (Table S9), and two biomarkers (*Barnesiella* and *Chlamydia*) were obtained to distinguish COVID‐19 baseline from health group (Figure S6C). To assess the correlation between fecal microbiota and COVID‐19 severity, COVID‐19 group was divided into mild and moderate/severe, using health group as baseline. Bacteria responsible for COVID‐19 severity included *E. coli*, *Burkholderiales* bacterium RIFCSPHIGHO2_12_FULL_63_20, *Actinomyces oris*, *Streptococcus parasanguini*, *Gemmiger formicilis*, and *Eisenbergiella tayi*. In general, all bacteria negatively related to COVID‐19 severity originated from *Bacteroides* (e.g., *Bacteroides thetaiotaomicron*, *Bacteroides caccae*, and *Bacteroides fragilis* (Table 2).

**TABLE 2:** Intestinal bacteria associated with COVID‐19 severity

Correlation	Taxon	Effect size	*p* value
Positive correlation with COVID‐19 severity	*Escherichia coli*	9.29146161	0.006919713
	*Burkholderiales* bacterium RIFCSPHIGHO2_12_FULL_63_20	8.532380941	0.009121345
	*Actinomyces oris*	11.20102218	0.003590297
	*Streptococcus parasanguinis*	9.439693129	0.00656345
	*Streptococcus* sp. I‐P16	10.57657041	0.004424267
	*Gemmiger formicilis*	8.380467999	0.009651017
	*Subdoligranulum* sp. 4_3_54A2FAA	9.591940264	0.006218832
	*Bifidobacterium longum*	8.730505117	0.008479023
	*Schaalia odontolytica*	8.326466264	0.009847578
	*Eisenbergiella tayi*	10.76444729	0.004152488
	*Intestinibacillus* sp. Marseille‐P4005	12.4346555	0.002411765
Negative correlation with COVID‐19 severity	*Bacteroides thetaiotaomicron*	9.965361263	0.005456389
	*Bacteroides caccae*	9.128274265	0.00733727
	*Bacteroides fragilis*	9.445210179	0.006550593

### 2.4 Functional characteristics of gut microbiome in COVID‐19 group

**FIGURE 4:**
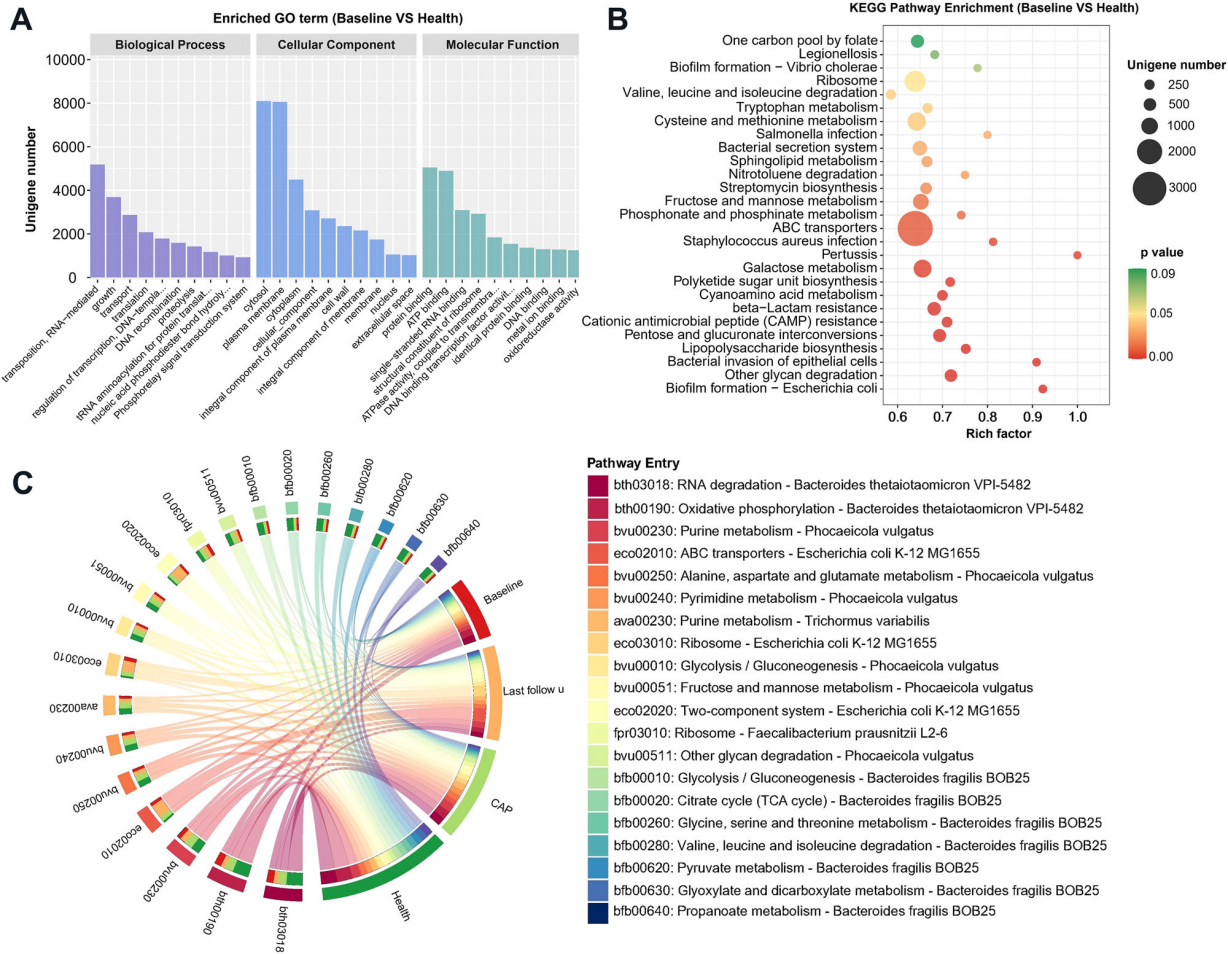
(A) Functional classification of differential genes upregulated in the COVID‐19 group according to gene ontology (GO) terms in the domains “molecular function” (MF), “cellular component” (CC), and “biological process” (BP). (B) Statistics of KEGG annotation of differential genes upregulated in the COVID‐19 group. The size of each circle represents the number of significant unigenes upregulated in the corresponding pathway (the significant threshold of differential genes as an absolute value of log2 (fold change) ≥1, *p* < 0.05). The upregulation factor was calculated with the number of upregulated gene divided by the total number of background genes in the corresponding pathway. A pathway with a *p* value <0.03 is considered significantly over‐represented. (C) Circos plot showing the information of most enriched pathways among microbiota in metagenome. Circos plots were divided into two parts. Leftmost part showed the pathway entry of gut microbiota based on annotation from KEGG database, while rightmost part represented four different cohorts. The leftmost part and rays (links) of circos are divided into 20 different colors according to the enrichment degree. The thickness of each ribbon represents the abundance from each cohort

Compared with health group, GO classification demonstrated the genes with RNA‐mediated transposition, growth, and transport were significantly upregulated in the biological process category. Genes related to the cytosol and plasma membrane were especially enriched in cellular component category. According to the molecular function category, genes involved in the protein binding, ATP binding, and single‐stranded RNA binding were significantly increased (Figure 4A). Apart from these, enrichment of oxidoreductase encoding genes was also observed. The metabolic pathways were remarkably altered in COVID‐19 group compared with the health group (Figure 4B). Most of them were relevant metabolism processing (namely, tryptophan metabolism; polyketide sugar unit biosynthesis; lipopolysaccharide biosynthesis; valine, leucine and isoleucine degradation sphingolipid metabolism; galactose metabolism, etc.), followed by human disease (*Staphylococcus aureus* infection; *Salmonella* infection; Pertussis, and Bacterial invasion of epithelial cells), environmental information processing (bacterial secretion system, ATP binding cassette (ABC) transporters), genetic information processing (CAMP resistance and β−Lactam resistance), and cellular processes (biofilm formation − *Escherichia coli* and *Vibrio cholerae*). Notably, significant change was found in energy metabolism of COVID‐19 group (Figure S7), and pathway entry also indicated that the butyrate synthesis pathway was remarkably lower than that in health group (Figure S8). Further, compared with CAP group, Kyoto Encyclopedia of Genes and Genomes (KEGG) pathways enriched in the COVID‐19 group were involved in opportunistic pathogen *Pseudomonas aeruginosa* and *S. aureus* infection (Figure S9). Subsequently, the differences of pathway entry among different cohorts were explored (Figure 4C). In *B. thetaiotaomicron* entry terms like RNA degradation and oxidative phosphorylation, health and CAP groups have more enriched genes than COVID‐19 cohort. However, the *E. coli* K‐12 MG1655 pathways (ABC transporters, ribosome and two‐component system) showed that the last follow‐up cohort accounts for a larger proportion, compared with baseline. It revealed that even in the COVID‐19 cohort, there are differences in MG statistics from the initial and subsequent stages of this disease.

### 2.5 Comparation of taxonomic and functional differences between MG and MT in COVID‐19 group

**FIGURE 5:**
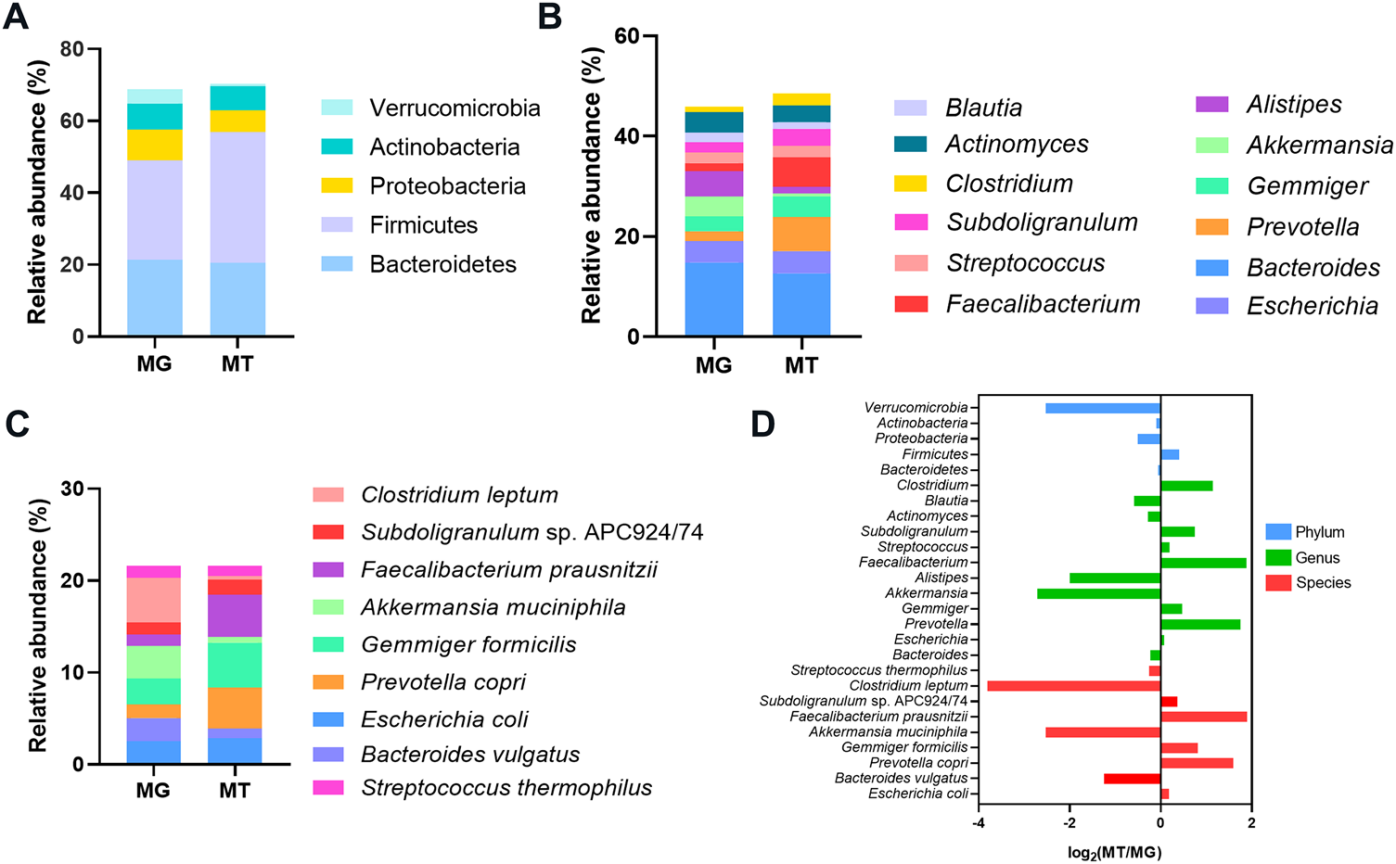
Gut microbiome composition of metagenome (MG) and metatranscriptome (MT) in COVID‐19 patients. (A–C) Bacterial composition at phylum, genus, and species levels (relative abundance ≥1%), respectively. Microbiota composition indicates the average relative abundance of bacterial presence in all samples (*n* = 10). (D) Ratio of mean relative abundance of microbes in MT to that in MG (MT/MG). Log2 is used for data normalization

To examine the potential activity of intestinal microbes detected in COVID‐19 patients, 10 COVID‐19 baseline fecal samples underwent MT sequencing, and an average of 17.17/16.78 giga row and clean reads was generated (Table S5). Five major phyla identified in MG (*Verrucomicrobia, Actinobacteria*, *Proteobateria*, *Firmicutes*, and *Bacteroidetes*) were also confirmed in MT (Figure 5A). At the level of genus, *Bacteroides* and *Escherichia* dominated both MG and MT data (Figure 5B). In terms of species level, *Gemmiger formicilis* were the main species (Figure 5C, Table S10). Next, we analyzed the ratio of the mean relative abundance in the MG to those in the corresponding MG (MT/MG ratio) to explore the relative activity of the baseline COVID‐19 microbiome (Figure 5D). The results demonstrated the relative activities of some butyrate producer bacteria, including *Blautia*,^22^ *C. leptum*, and *A. muciniphila* ^32^ were decreased, while *P. copri* ^33^ and *E. coli* displayed high transcriptional activity. It is noteworthy that a high MT/MG ratio of several bacteria negatively correlated with COVID‐19 (e.g., *F. prausnitzii, B. ovatus*, *B. fragilis*, and *B. caccae*) was observed (Table S11).

**FIGURE 6:**
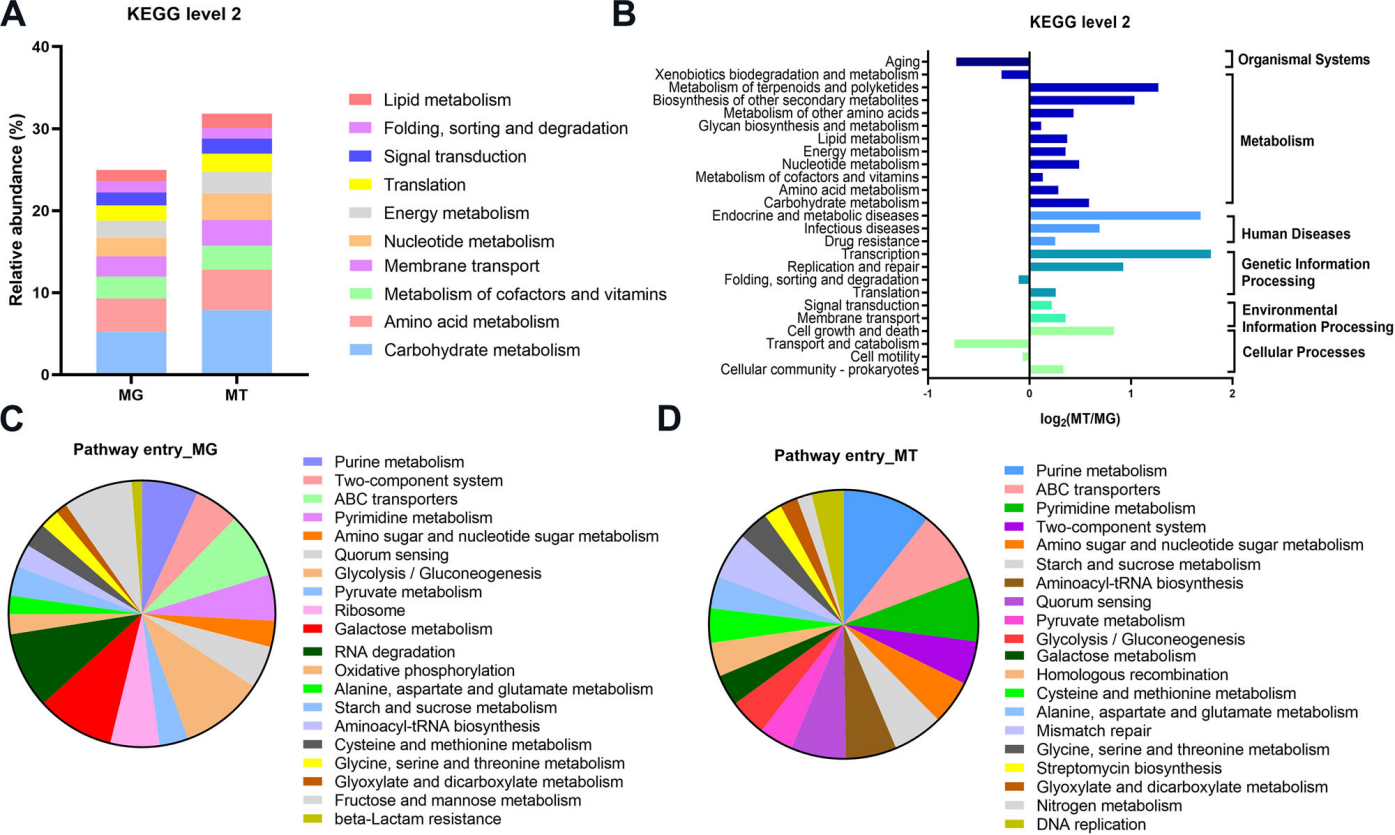
KEGG annotations of the intestinal metagenome and metatranscriptome in COVID‐19 patients (*n* = 10). (A) KEGG level 2 annotations of fecal metagenome (MG) and metatranscriptome (MT) data (Top 10 in abundance). (B) Ratio of mean relative abundance of KEGG level 2 annotations in MG to that in MT (MT/MG). The assignment of KEGG pathway entry annotations of MG (C) and MT (D) statistics

To functionally characterize the active gut microbiome of COVID‐19 patients, unigenes of the MG and MT were aligned to protein sequences from KEGG databases. In both the MG and MT data, “carbohydrate metabolism,” “Amino acid metabolism,” and “Metabolism of cofactors and vitamins” were most enriched KEGG pathways (Figure 6A). The pathways related with human diseases such as “drug resistance,” “infectious diseases,” and “endocrine and metabolic diseases” were upregulated (Figure 6B). At metabolic‐related modules, pathways including “energy metabolism” and “amino acid metabolism” were also actively expressed. However, “xenobiotics biodegradation and metabolism” showed downregulated expression levels. More fined grained modules uncovered that purine metabolism is the most important pathway entry (Figure 6C,D). Further, the ratio of MT to MG also demonstrated the active expression of ABC transporter and beta‐lactam resistance metabolic pathways (Figure S10). As mentioned earlier, these pathways may imply upregulation of toxic stress.^34^

### 2.6 Correlations between clinical indicators and fecal bacteria in COVID‐19 group

We identified the correlation between gut microbiota (COVID‐19) and clinic statistics by Pearson analysis (Figure S11A,B). The MG data results exhibited that *Bacteroides stercoris, Bacteroides vulgatus*, and *Alistipes* sp. HGB5 are positively correlated with infectious indicator procalcitonin (PCT), while *Alistipes* sp. HGB5 is negatively associated with indicators of liver function including alanine aminotransferase (ALT), aspartate aminotransferase (AST), and lactate dehydrogenase (LDH). Interestingly, there is a negative correlation between *B. thetaiotaomicron* and coagulation indicator prothrombin time (PT). The MT data showed that *B. thetaiotaomicron* is positively interacted with neutrophil (NEUT) and eosinophils (EOS), suggesting its beneficial effect in immune modulation. Apart from that *S. odontolytica* and *Actinomyces* sp. ICM58 are both negatively associated with platelet (PLT) and globulin, further testified the causal relationship between *S. odontolytica* and the severity of COVID‐19 patients. Notably, among the nine bacteria positively correlated with PCT, *Firmicutes* bacterium CAG:5, *Firmicutes* bacterium CAG:103, and *C. leptum* are positively associated with coagulation indicators D‐dimer and PT.

## 3 DISCUSSION

The change characteristic of lymphocyte in COVID‐19 patients was consistent with previous epidemiological researches,^35^, ^36^ reflecting the possible bacterial infection and immune response of COVID‐19 patients exposed to SARS‐CoV‐2 virus.^37^, ^38^ Shorter APTT is often related to elevated risk of hypercoagulability and thromboembolism.^39^ The clot waveform of APTT also suggested that COVID‐19 patients might have distinctive abnormal coagulopathy.^40^ High‐level TIBIL is generally considered a marker of abnormal liver metabolism and hepatitis,^41^ and there appears to be a significant relationship between HGB levels and COVID‐19 disease severity.^42^ These results suggested that enrolled COVID‐19 patients may have liver damage, pathogen infection, and blood system disorder.^43^

Compared with healthy people, the gut microbiota structure of COVID‐19 patients has changed significantly. Even after lung/gut viral clearance, the intestinal microbiota structure in COVID‐19 patients has not returned to normal. In addition, depletion of symbiotic bacteria has been observed in the baseline gut microbiota of COVID‐19 patients, including *B. vulgatus*, *C. leptum*, and *A. putredinis*. *B. vulgatus* exhibited the effective inhibition of proinflammatory immune response in atherosclerotic lesions.^44^ Its specific colonization was also identified as a low‐risk predictor of immune‐related adverse events in metastatic melanoma.^45^ Previous works indicated that *Alistipes* spp. not only was reduced in COVID‐19 patients but also negatively correlated with COVID‐19 severity,^22^, ^46^, ^47^ which may be contributed to its participation in tryptophan metabolism and protective role in intestinal immune homeostasis.^48^ Another study also observed significant decrease in the abundance of butyrate producer *C. leptum*.^22^ It had been shown that the decline of the butyrate producer is not conducive to COVID‐19 recovery.^21^, ^22^, ^49^ As one of the most important energy metabolism substrates for intestinal microbiota, butyrate plays a positive role in maintaining mucosal barrier, providing antiviral immune response and reducing inflammation.^50^ On the other hand, *E. coli* was significantly amplified in COVID‐19 baseline patients. As is well‐known, partial subspecies of *E. coli* are important pathogens causing a variety of intestinal and parenteral infections.^51^ It is surprising that the increased abundance of *A. muciniphila* existed in COVID‐19 baseline samples. *A. muciniphila* can improve intestinal barrier and provide host immune responses.^52^ Even so, research had proved that its abundance is positively correlated with H7N9 infection and disease severity.^53^ This might be due to the increased levels of Muc2,^54^ an essential ingredient for the growth of this bacterium,^55^ caused by respiratory virus infection.^56^ However, oral administration of *A. muciniphila* still inhibited H7N9 proliferation and improved clinical symptoms in C57BL/6 mice experiment.^57^ Thereby, the endogenous increase of *A. muciniphil* in COVID‐19 might be harmless.

Subsequently, LEfSe analysis identified species differences between COVID‐19 patients and control groups. The specially enrichment of *S. odontolytica* in baseline gut microbiota of COVID‐19 group increased the risk of bacteremia.^58^, ^59^ Besides, *S. odontolytica* was discovered in various pulmonary infections, which may be associated with the development of acute respiratory distress syndrome.^60^, ^61^ *Lactobacillus rhamnosus* is depicted as a potential co‐infection microorganism along with SARS‐CoV‐2.^62^ *Lactobacillus* had been reported to aggravate mucosal inflammation, and interestingly, lactic acid was rich in fecal samples from COVID‐19.^30^ In the last follow‐up sample, several opportunistic pathogens were characteristic species for COVID‐19 patients. Specifically, *K. pneumoniae* is common lung pathogen, and both *S. dysenteriae and S. flexneri* are notorious gastroenteritis triggers.^63^, ^64^ Some opportunistic pathogens have also been found to be positively associated with the COVID‐19 severity. Among them, *Streptococcus* was enriched in high SARS‐CoV‐2 feature fecal samples from human^21^, ^65^ or primate.^66^ The finding that *A. oris* related to COVID‐19 severity further ascertains the association between *Actinomyces* spp. with the progression of SARS‐CoV‐2 gastrointestinal infection.^21^, ^22^, ^67^ *E. tayi* increases the risk of bacteremia.^65^, ^66^ In addition, *Burkholderiales* spp. has been associated with inflammatory bowel disease.^70^ In contrast, several specific *Bacteroides* have been identified as potentially blocking the COVID‐19 process. Consistent with the previous study, decreased abundance of *Bacteroides* was positively correlated with disease severity.^23^, ^47^, ^66^ *B. thetaiotaomicron* could downregulate ACE2 expression level in colon.^22^, ^68^ Moreover, it was metabolically complementary to butyrate‐producing bacterium *F. prausnitzii* ^69^ and could together modulate the intestinal mucus barrier to reduce SARS‐CoV‐2 virus load.^22^, ^23^ On the other hand, *B. fragilis* was involved in antiviral defense by inducing colonic plasmacytoid dendritic cells^68^; whereas, *B. caccae* could regulate gut IgA levels.^70^ In addition, *Bacteroides* could digest dietary and polysaccharides as host energy sources to promote the immune system.^71^

The enriched oxidoreductase activity in COVID‐19 group hinted that active gut microbiota promoted energy‐yielding biochemical reactions.^72^ Protein binding, single‐stranded RNA binding and structural constituent of ribosome showed close relationships to metabolic processes.^73^ Enrichment of pathogens relative pathways (i.e., *Staphylococcus aureus* infection, *Salmonella* infection, Pertussis, and Bacterial invasion of epithelial cells) indicated the human gut is the site of extrapulmonary bacterial infection. Highly active expression of *E. coli* and upregulation of genes related to biofilm formation supported their association with COVID‐19 severity. Galactose utilization could result in hypervirulent phenotype of *Streptococcus pneumoniae*.^74^ Dysregulation of the ABC transporter pathway implies that patients might be under toxic stress after exposure to SARS‐CoV‐2.^34^ Besides, the accessory genomes of considerable pathogenic bacteria cover ABC transporters that contribute to antimicrobial resistance by multidrug efflux,^75^ further explaining antibiotic resistance pathway enrichment. Sphingolipids are progressively recognized as critical mediators in participation with inflammatory responses and multiple pulmonary diseases.^76^ The biosynthesis of polyketide sugar unit and lipopolysaccharide could be related to oxidative stress state and risk of microbial translocation to systemic inflammation, respectively.^77^ The results of pathway annotation also indicated abnormal energy metabolism of intestinal microbiota in the COVID‐19 group. Notably, impaired butyrate synthesis may indicate nutrient deficiencies in host cells. Furthermore, the increased neutral amino acids degradation and tryptophan metabolism may be related to the consumption of ACE2, because the ACE2 is involved in tryptophan uptake^11^ and closely related to the expression of the amino acid transporter B0AT1.^78^ Intriguingly, branched‐chain fatty acids derived from neutral amino acids degradation were related to obesity, metabolic syndrome, and diabetes.^79^ Taken together, intestinal microbiota impacted COVID‐19 virulence, further participating in the pathophysiology of the host.

Lastly, as indicated from the MT data, the COVID‐19 patients had various metabolically active microbiota. Among them, phylum *Verrucomicrobia* exhibited low transcriptional activity, which may be attributed to the low active state of *A. muciniphila*. The anti‐inflammatory effect of *A. muciniphila* depends on the outer membrane protein Amuc_1100.^80^ Such a result may partially explain why the increased abundance of *A. muciniphila* did not endogenously alleviate COVID‐19 and H7N9 progressions.^57^ *C. leptum* was reduced in the COVID‐19 group, and its low metabolic activity further impeded its positive effect on disease progression. The active metabolism of *P. copri* might also adversely affect COVID‐19 development. In the upper respiratory tract, *Prevotella* was found to be positively associated with SARS‐CoV‐2 viral load.^33^ Moreover, higher abundance of *P. copri* in gut is correlated with lower risks of systemic inflammation^81^ and human immunodeficiency virus infections.^82^ As mentioned earlier, in MT data, the highly active “cell wall/membrane/envelope biogenesis” pathways participated in bacterial biofilm formation under SARS‐CoV‐2 infection, especially in *E. coli*. Moreover, active “membrane transport” might be associated with antimicrobial resistance excretion.

The infection of SARS‐CoV‐2 not only consumes a lot of energy in the host cell,^20^ but also reduces ACE2 expression, thus damaging intestinal epithelial cells and affecting transporter B0AT1 functions, which leads to intestinal barrier disruption, amino acid starvation, ion imbalance, and immune inflammatory environment.^11^, ^83^ Compared with healthy controls, the composition of gut microbiota in COVID‐19 patients has undergone profound alterations, including significant reduction in diversity, enrichment of pathogens, and the consumption of commensals. Notably, this study reaffirmed that *Bacteroides* might play an essential role in mitigating COVID‐19 progression.^22^, ^23^, ^67^ Moreover, the gut microbiota function of COVID‐19 patients is extraordinarily different from that of health group: enhanced metabolism of neutral amino acids, abnormal energy metabolism, high oxidative stress, and excessive inflammation responses. Specially, the malfunction of butyrate synthesis may suggest its adverse effect in disease progression.^22^, ^23^ In turn, healthy microbiome can influence the course of COVID‐19 disease development by enhancing viral colonization resistance, producing beneficial bacterial metabolites, and triggering local immune recalibration. Some limitations of the study should be mentioned. First, this is a single‐center study with a moderate sample size, which does not apply to all COVID‐19 patients. The corresponding relationship between SARS‐CoV‐2 infection and intestinal microbiota dysbiosis should be validated in a larger cohort, including subgroups at different stages of the disease. Though several species that may be central players for COVID‐19 progression were discussed and reviewed (Table S12 and S13), meta‐analysis from current multicenter and different studies to obtain universal conclusions is urgently warranted.^21^, ^22^, ^23^, ^30^, ^47^, ^67^, ^84^ Moreover, this study depicted the alterations between patients at different stages of COVID‐19, while no specific assessment has been conducted on the changes in COVID‐19 microbiota and functions over time. Albeit we tried to control the variation degree between COVID‐19 patients and the healthy controls, the alternations of gut microbiota may be influenced by other confounding factors, such as lifestyle, dietary habits, underlying diseases, complications, and clinical management. Lastly, the disease stage of COVID‐19 at the time of stool sample collection is uncertain, and there is a lack of information on preinfection stool samples.

Collectively, this study further revealed alterations in the composition and function of active intestinal microbiota in COVID‐19 cases. Specific microbial biomarkers of COVID‐19 patients were screened and correlated with clinical indicators. These results may deepen our understanding of how SARS‐CoV‐2 interferes with gut microbes and provide a treatment option for the fine‐tuning gut microbiome in addition to the COVID‐19 conventional treatment regimens.

## 4 MATERIALS AND METHODS

### 4.1 Epidemiological investigation

During the COVID‐19 pandemic in China, all highly suspicious and confirmed cases in Lanzhou (36°03′ N, 103°40′ E) were admitted to hospitals abiding by the infectious disease law. The First Hospital of Lanzhou University performed nasopharyngeal swabs‐based qRT‐PCR to screen 836 suspected patients to confirm SARS‐CoV‐2 infection. A total of 13 cases of COVID‐19 and 24 cases of CAP were hospitalized. The patients diagnosed with SARS‐CoV‐2 infection by virology laboratory of this hospital were further confirmed by Lanzhou center for disease control and prevention (CDC) or Gansu provincial CDC. COVID‐19 patients were classified as mild, moderate, or severe according to disease progression severity.^22^ All pneumonia cases were recruited to this study for epidemiological investigation. Thirteen healthy subjects with age, body mass index (BMI), and gender matching COVID‐19 patients were also enrolled. Patients were cross‐examined by hospital staffs pursuant to standardized questionnaires to generate clinical presentations and demographics. Medical records and laboratory results were reviewed to collect data on chest computerized tomography (CT), blood routine examination (leukocyte/white blood cell [WBC], neutrophil [NEUT], lymphocyte [LYM], platelet [PLT], eosinophils [EOS], basophils [BAS], hemoglobin [HGB], globulin), coagulation function (activated partial thromboplastin time [APTT], D‐dimer, prothrombin time [PT], fibrinogen), biochemical indicators (aspartate aminotransferase [AST], alanine aminotransferase [ALT], total bile acid [TBA], total bilirubin [TBIL], uric acid [UA], lactate dehydrogenase [LDH], α‐hydroxybutyric dehydrogenase [α‐HBDH], glucose), and inflammatory biomarkers (C‐reactive protein [CPR], procalcitonin [PCT], creatine kinase [CK]). Examination of viral excretion from the nasopharyngeal and fecal samples was conducted by serial qRT‐PCR.

### 4.2 Feces sampling and DNA/RNA extraction

The fresh fecal samples were collected by hospital staff using fecal collection tubes and a sterile stick, including 20 COVID‐19 patient samples (COVID‐19 group, including 13 baseline samples and seven progression samples) (Figure 1), 13 healthy samples (health group), and eight CAP samples (CAP group). All baseline samples of COVID‐19 were collected during hospitalization (Figure 1). Each fresh sample was delivered immediately from the ward to virology laboratory with ice packs, where it was divided into aliquots of 1 g and frozen in liquid nitrogen, stored at −80°C until the next step. Next, DNA from all the above collected samples was extracted, and RNA was extracted from 10 baseline fecal samples of COVID‐19 group (Figure S1). In brief, total bacterial deoxyribonucleic acid (DNA) was extracted from the frozen aliquot of each fecal sample by using E.Z.N.A. stool DNA kit (Omega, USA) following the manufacturer's instruction. One percent agarose gel electrophoresis was employed to estimate DNA integrity. DNA purity was measured using nanodrop spectrophotometer (Thermo Scientific, USA), and its concentration was determined using Qubit quantification system (Thermo Scientific, Wilmington, DE, USA). Total RNA was isolated and purified using E.Z.N.A. stool RNA kit (R6828, Omega, USA) following the manufacturer's procedure. The RNA amount and purity of each sample were quantified using NanoDrop ND‐1000 (NanoDrop, Wilmington, DE, USA). The RNA integrity was assessed by Agilent 2100 with RIN number >7.0. The DNA/RNA that conforms sequencing requirements was then stored at −80/°C (Figure S1).

### 4.3 MG and MT sequencing and data analysis

DNA library was constructed by TruSeq nano DNA LT library preparation kit (FC‐121‐4001). In brief, DNA was fragmented by dsDNA Fragmentase (NEB, M0348S). The cDNA library was constructed by repairing the end of the DNA fragment, adding “A” base to the blunt ends of each strand, adding sequencing adapters, fragments selection, and PCR amplification. For the extracted RNA samples, the Ribo‐Zero rRNA removal kit (Illumina, San Diego, USA) was adopted to deplete rRNA and other host RNA sequences from total RNA. Subsequently, the left RNAs were fragmented and reverse‐transcribed into cDNA. The cDNA library for sequencing was constructed as described in the above description. Finally, all cDNA libraries were sequenced on Illumina Novaseq 6000 (LC Bio, China) (Figure S1). The sequencing mode was performed with 150 bp paired end. Sequencing adapters and low‐quality reads were filtered and trimmed from raw sequencing data by using cutadapt v1.9 and fqtrim v0.94 (sliding‐window algorithm), respectively. Next, qualified reads were aligned to the human genome by employing Bowtie2 v2.2.0 to remove host contamination, followed by de novo assembly to construct the contigs for each sample by respectively applying IDBA‐UD v1.1.1 and Trinity v2.2.0. All coding regions of contigs were predicted by using MetaGeneMark v3.26. And then, the contigs were clustered to by CD‐HIT v4.6.1 to obtain unigenes. Transcripts per kilobase million (TPM) was used to estimate the unigene abundance of a certain sample according to the aligned reads number of Bowtie2 V2.2.0. Then, unigenes were aligned against the National Center for Biotechnology Information (NCBI) Non‐Redundant Protein Sequence Database (NR) database to obtain the lowest common ancestor taxonomy of them with DIAMOND v 0.9.14. Likewise, the GO/KEGG annotations of unigenes were obtained.

### 4.4 Statistical analyses

The characteristics of the COVID‐19 patients were described through demographics, epidemiological data, clinical signs and symptoms on admission, chest radiographic findings, laboratory results, treatment, and clinical outcomes. Alpha diversity was calculated using QIIME v1.8.0. The principal coordinate analysis (PCoA) based on Bray‐Curtis distance was used to assess beta diversity. The similarity analysis (ANOSIM) and permutational multivariate analysis of variance (PERMANOVA/Adonis) were conducted to compare the difference of microbiota structure between groups. The Adonis analysis was also performed to compare the effect size of subject metadata on microbiota composition. Using ANOVA with Tukey multiple test correction to evaluate microbiome related to COVID‐19 severity (*p* < 0.01 was considered significant and F value was used as effect size). Differential species between groups were identified conducting LEfSe analysis, and taxa with an LDA score > 3.0 were considered significantly different. Pearson test was employed to evaluate the correlations between clinical indexes and COVID‐19 bacteriome and only correlations with a statistically significant value (*p* < 0.05) were marked with an asterisk symbol. All *p* values deriving from correlograms, LEfSe was adjusted by Benjamini–Hochberg false discovery rate (FDR) correction to obtain *q* values (adjusted *p* values). Finally, Kruskal–Wallis test with Dunn’s multiple comparison test was used to analyze the differences of clinical data, taxa/GO annotated genes/KEGG pathway between groups.

## CONFLICTS OF INTEREST

The authors have no conflict of interest to declare.

## ETHICS STATEMENT

All participants provided written informed consents prior to starting the study. Research protocols were approved and supervised by the Institutional Review Board of the First Hospital of Lanzhou University and conformed to the ethical guidelines of the 1975 Declaration of Helsinki (Serial number: LDYYLL2020‐24).

## AUTHOR CONTRIBUTIONS

Tuoyu Zhou: Conceptualization; Writing – original draft; Validation and Formal analysis. Jingyuan Wu: Writing – original draft; Validation and Formal analysis. Yufei Zeng: Genomic data analysis. Junfeng Li: Stool samples collection. Jun Yan: Epidemiological investigation. Wenbo Meng: Epidemiological investigation. Huawen Han: Revision. Pengya Feng: Methodology. Jufang He: fecal DNA and RNA extraction. Shuai Zhao: Stool samples collection. Ping Zhou: fecal DNA and RNA extraction. Ying Wu: Sample collection. Yanlin Yang: fecal DNA and RNA extraction. Rong Han: Epidemiological data curation. Weilin Jin: Revision. Xiangkai Li, Yunfeng Yang and Xun Li: Supervision and Funding acquisition.

## Supporting information

Supporting information

## Data Availability

All the sequences supporting the results of the current study have been deposited in the National Center for Biotechnology Information (NCBI) Sequence Read Archive (SRA) under BioProject accession number PRJNA740067.
