# Supplementary material for: SARS‐CoV‐2 triggered oxidative stress and abnormal energy metabolism in gut microbiota
Source: MedComm (2020). 2022 Jan 17;3(1):e112. doi: 10.1002/mco2.112 (PMC8906553; doi:10.1002/mco2.112)
Supplement: Supplementary file 1 — Supporting information [file MCO2-3-0-s001.docx]

**Supplementary Information**

**Tuoyu Zhou^1^#**, **Jingyuan Wu^2^#**, **Yufei Zeng^3^#, Junfeng Li^2^#**, Jun Yan^2^, Wenbo Meng^2^, Huawen Han^1^, Fengya Feng^1^, Jufang He^2^, Shuai Zhao^1^, Ping Zhou^2^, Ying Wu^1^, Yanlin Yang^2^, Rong Han^1^, Weilin Jin^4^, **Xun Li^2^***, **Yunfeng Yang^3^***, **Xiangkai Li^1^***

1. Ministry of Education Key Laboratory of Cell Activities and Stress Adaptations, School of Life Sciences, Lanzhou University, Lanzhou, Gansu 730000, P. R. China
2. Gansu Province Key Laboratory Biotherapy and Regenerative Medicine, The First Hospital of Lanzhou University, Lanzhou, Gansu 730000, P. R. China
3. State Key Joint Laboratory of Environment Simulation and Pollution Control, School of Environment, Tsinghua University, Beijing 100000, P. R. China
4. Medical Frontier Innovation Research Center, The First Hospital of Lanzhou University, Lanzhou, Gansu 730000, P. R. China

**#**These authors contributed equally to this work: **Tuoyu Zhou**, **Yufei Zeng**, **Jingyuan Wu**, **Junfeng Li**

***Corresponding author:**

Prof. Xun Li, Ministry of Education Key Laboratory of Cell Activities and Stress Adaptations, School of Life Sciences, Lanzhou University, Lanzhou, Gansu, P. R. China. Email: [lxdr21@126.com](mailto:lxdr21@126.com). Fax: 0931-8619797

Prof. Yunfeng Yang, State Key Joint Laboratory of Environment Simulation and Pollution Control, School of Environment, Tsinghua University, Beijing, P. R. China.

Email: [yangyf@tsinghua.edu.cn](mailto:yangyf@tsinghua.edu.cn). Tel: 010-62784692

Prof. Xiangkai Li, Gansu Province Key Laboratory Biotherapy and Regenerative Medicine, The First Hospital of Lanzhou University, Lanzhou, Gansu, P. R. China. Email: [xkli@lzu.edu.cn](mailto:xkli@lzu.edu.cn); Tel: +86-931-8912560; Fax: +86-931-891256

**Content**

**Supplemental Figures**

**Figure S1** Flowchart illustrates the recruitment of COVID-19 patients, healthy controls and community-acquired pneumonia (CAP) cases based on inclusion criteria.

**Figure S2** Comparation of clinical biomarkers between COVID-19, heath and CAP groups.

**Figure S3** The effect size of subject metadata, analyzed by adonis.

**Figure S4** Beta diversity plot of baseline stool samples based on principal coordinates analysis (PCoA) of Bray–Curtis distance, with or without antibiotic exposed.

**Figure S5** Inter-individual dissimilarities between fecal microbiota within COVID-19, heath and CAP groups.

**Figure S6** The random forest model based on genus-level abundance taxa.

**Figure S7** KEGG Level 2 annotation statistics between COVID-19, Health and CAP groups.

**Figure S8** Butyrate synthesis pathway based on KEGG pathway entry annotation. **Figure S9** KEGG annotation statistics of different genes between COVID-19 group and CAP group.

**Figure S10** Ratio of mean relative abundance of KEGG pathway entry annotations in MG to that in MT.

**Figure S11** Relationship between clinical information of COVID-19 and species composition based on MG and MT data.

**Supplemental Tables**

**Table S1** Clinical characteristics of n = 13 healthy controls

**Table S2** Clinical characteristics of n = 13 independent patients confirmed with SARS-CoV-2 infection

**Table S3** Clinical characteristics of n = 24 independent patients with community-acquired pneumonia

**Table S4** MG sequence statistics of COVID-19, health and CAP groups

**Table S5** MT sequence statistics of COVID-19 patients

**Table S6** PERMANOVA and ANOSIM test for pairwise comparisons among the Baseline, Last follow up, Health and CAP groups

**Table S7** Gut microbial composition of different cohorts based on MG data

**Table S8** Pair comparison of intestinal microbiota composition in different cohorts

**Table S9** Gut microbes feature in COVID-19 and CAP patients based on LEfSe analysis

**Table S10 G**ut microbial composition of COVID-19 patients based on MT data

**Table S11** Ratio of bacterial average relative abundance between MT and MG

**Table S12** Gut bacteria (genus and species) with altered abundance in COVID-19 patients

**Table S13** Gut bacteria (genus and species) associated with COVID-19 severity


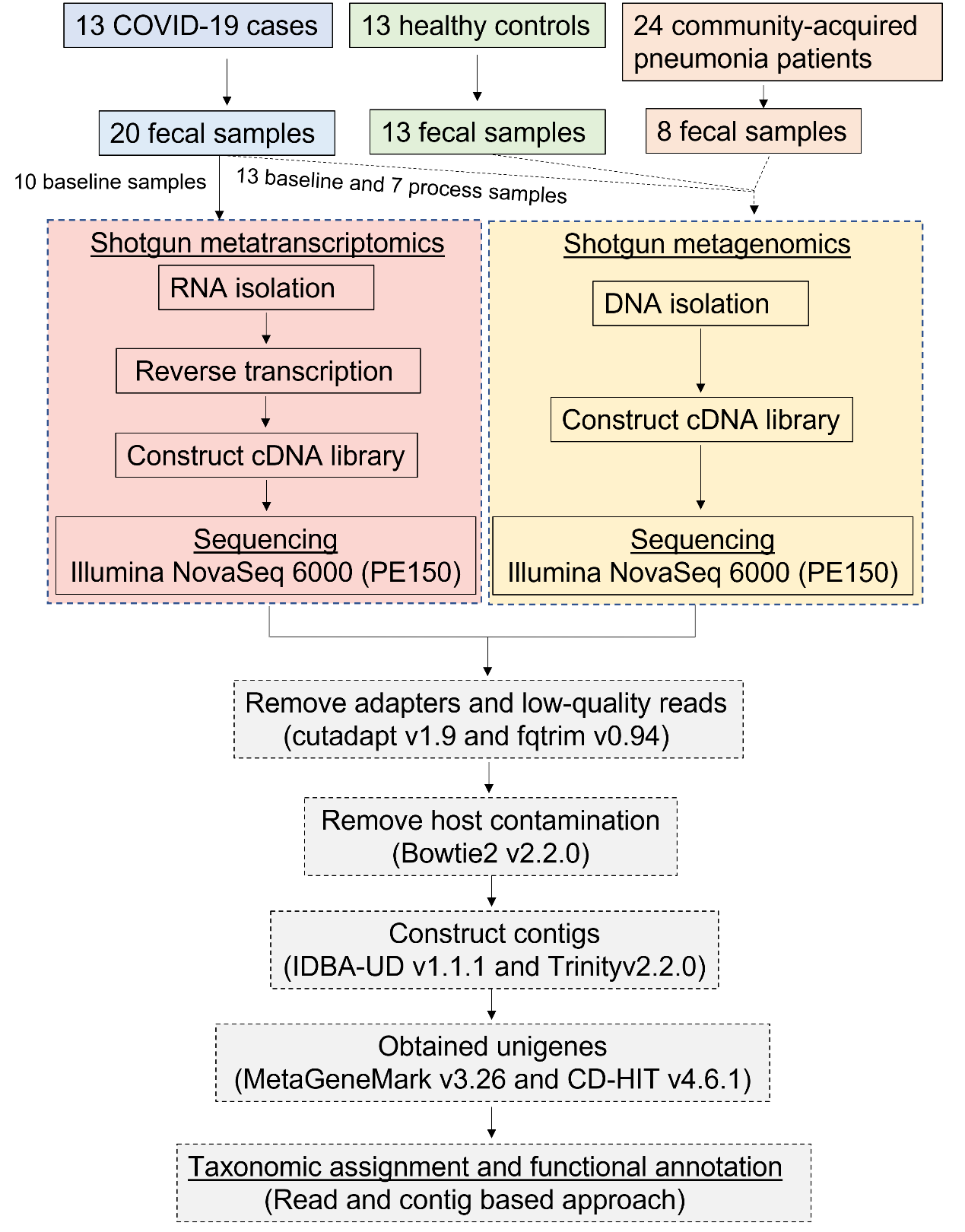
**Supplementary Figure 1** Flowchart illustrates the recruitment of patients with COVID-19 group, healthy controls and community-acquired pneumonia cases based on inclusion criteria.


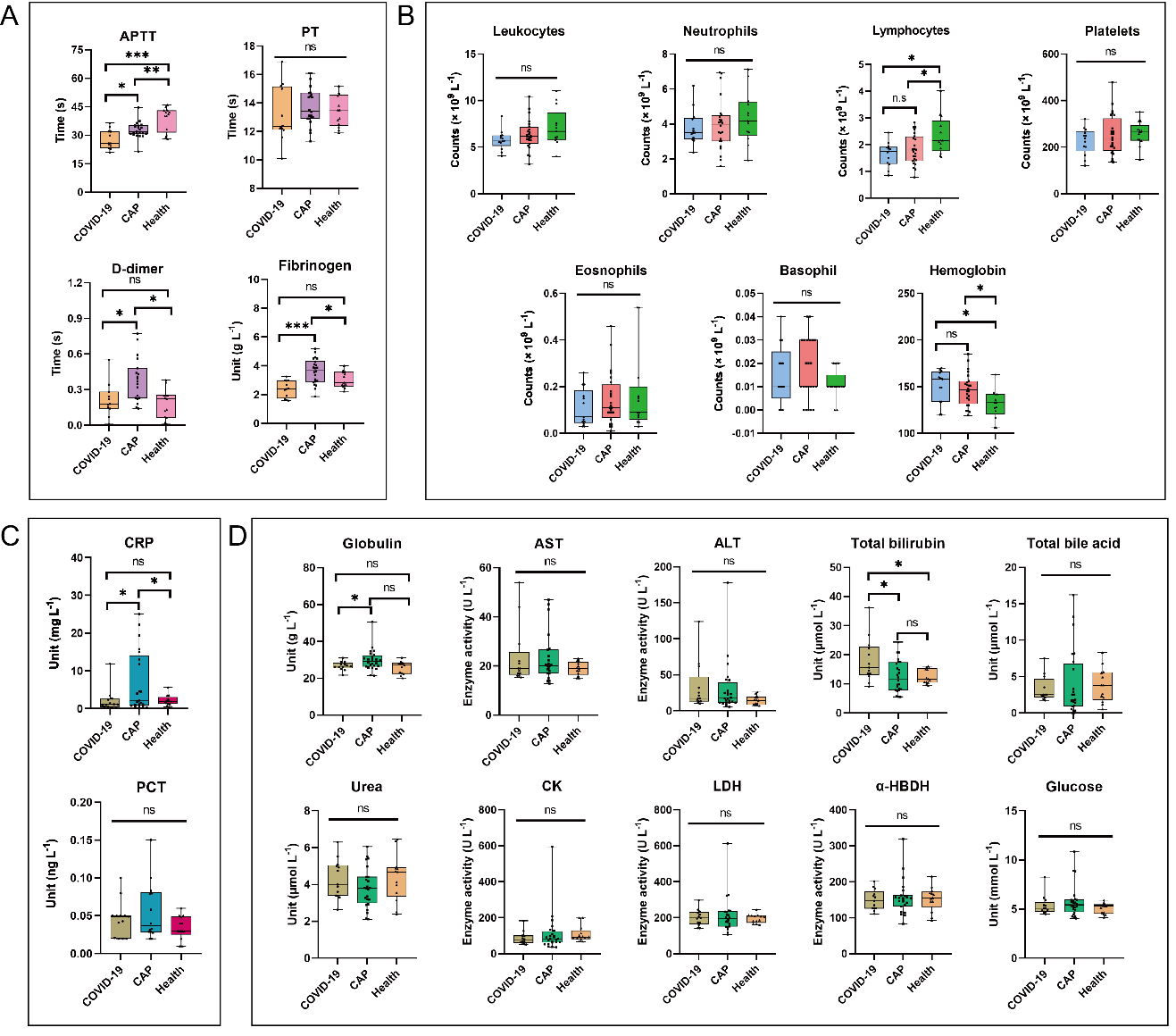


**Supplementary Figure 2** Comparation of clinical biomarkers between COVID-19 (n=13), heath (n=13) and CAP (n=25) groups, including (A) coagulation parameters; (B) blood routine results; (C) infectious criterions and (D) biochemistry indexes. PT, prothrombin time; APTT, activated partial thromboplastin time; CRP, C-reactive protein; PCT, procalcitonin. AST, aspartate aminotransferase; ALT, alanine aminotransferase; CK, creatine kinase; LDH, lactate dehydrogenase; α-HBDH, α-hydroxybutyric dehydrogenase. Significance was marked as **p* < 0.05, ***p* < 0.01, ****p* < 0.001.


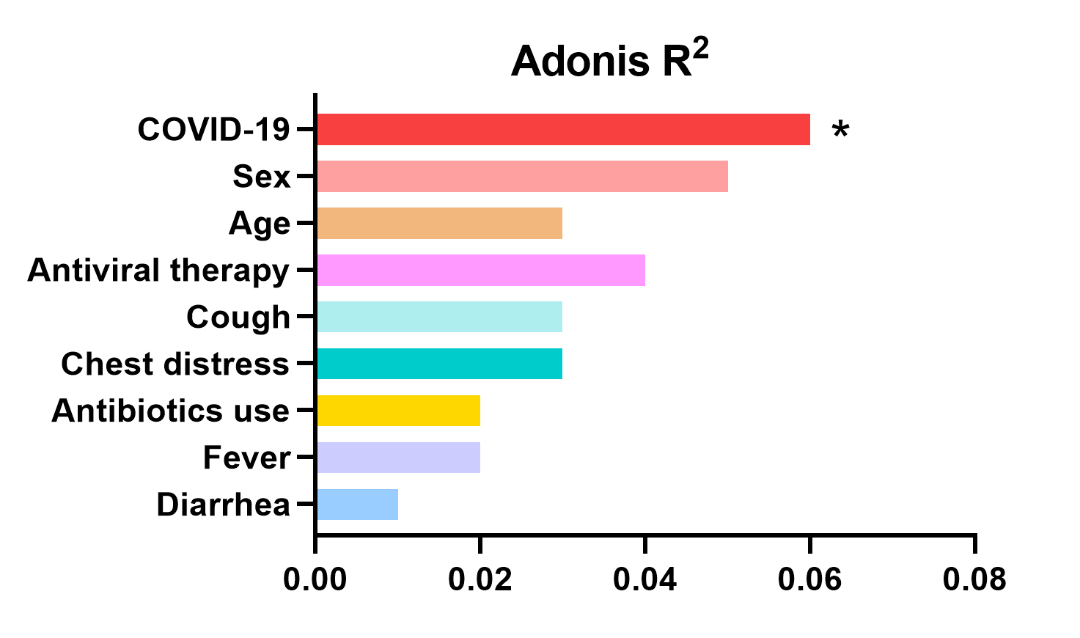


**Supplementary Figure 3** The effect size of subject metadata, analyzed by Adonis. Significance was marked as **p* < 0.05, ***p* < 0.01, ****p* < 0.001.


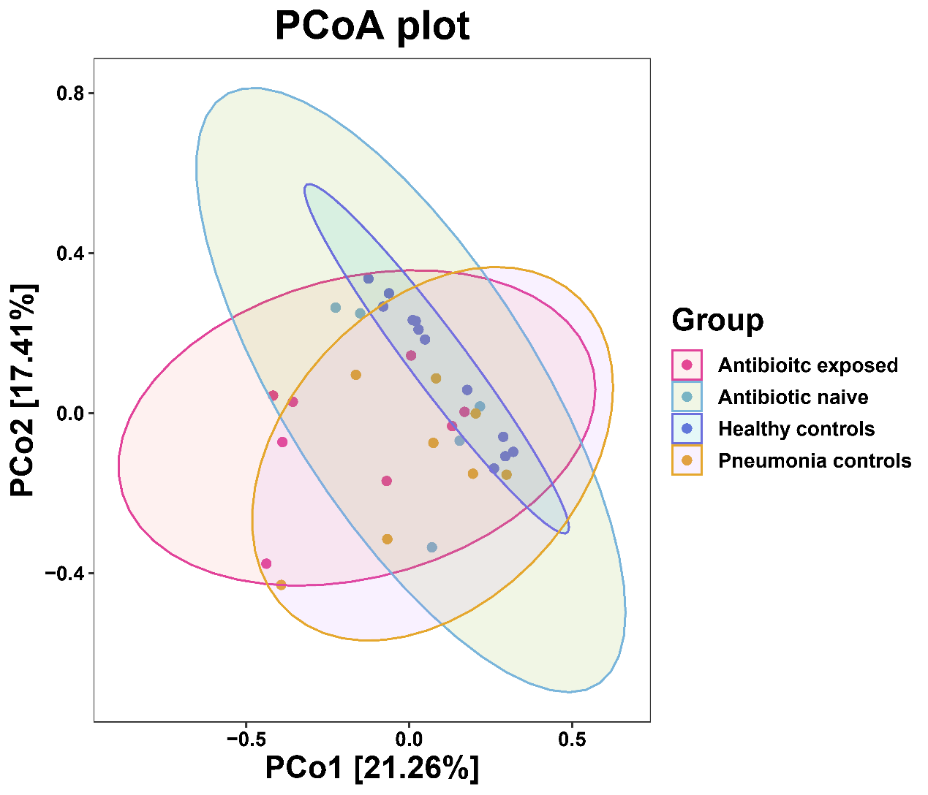


**Supplementary Figure 4** Beta diversity plot of baseline stool samples based on principal coordinates analysis (PCoA) of Bray–Curtis distance. Red and green represent the antibiotic exposed and antibiotic naïve group in COVID-19, while blue and orange indicated community-acquired pneumonia and healthy group, respectively. ANOSIM analysis showed significant differences between both antibiotic exposed and antibiotic naïve groups (R = 0.34 and -0.07, *p* = 0.01 and 0.74, respectively), but no significant differences between the two groups (R = -0.15, *p* = 0.95).


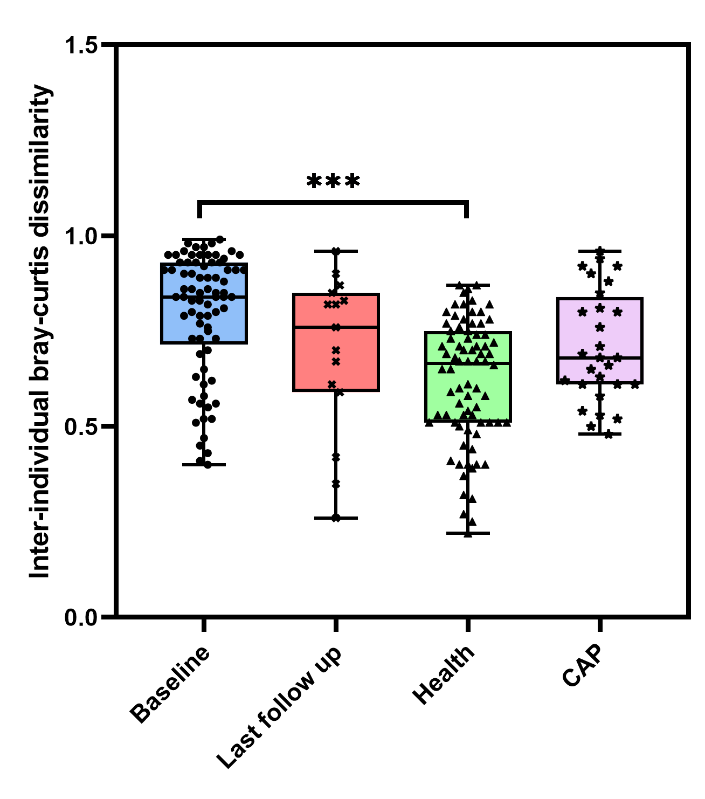


**Supplementary Figure 5** Inter-individual dissimilarities between fecal microbiota within each group. Between-group comparison was conducted by Kruskal-Wallis test. Significance was marked as **p* < 0.05, ***p* < 0.01, ****p* < 0.001.


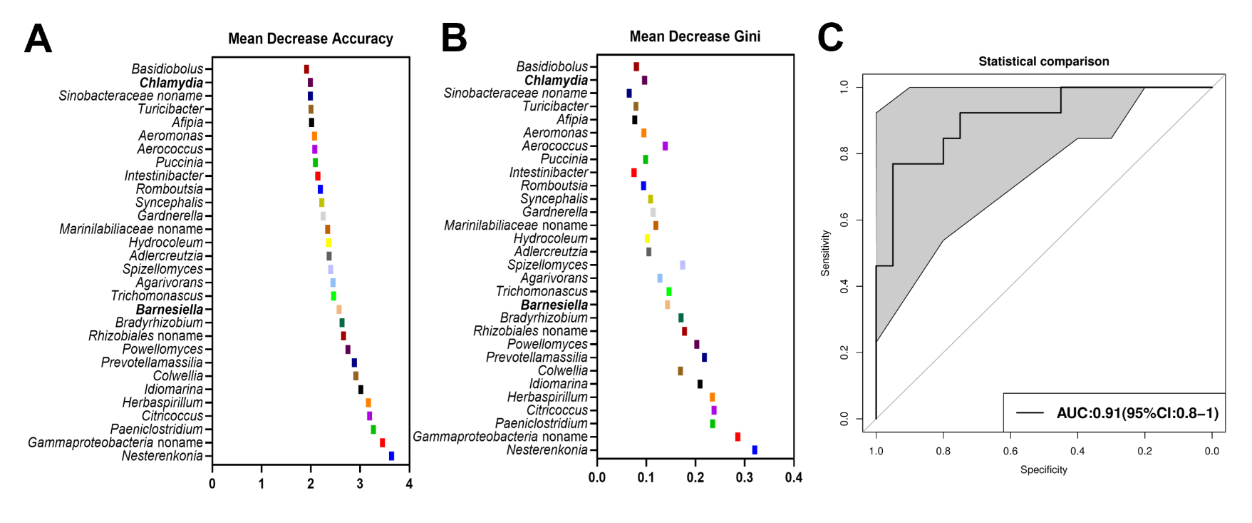


**Supplementary Figure 6** The random forest model based on genus-level abundance taxa. The relative importance of each genus in the predictive model was performed using the mean decreasing accuracy (A) and the Gini coefficient (B) for fecal microbiota. (C) Prediction of two biomarkers (*Barnesiella* and *Chlamydia*) in the microbiota of COVID-19 and healthy controls. The area under the ROC curve is displayed in the center.


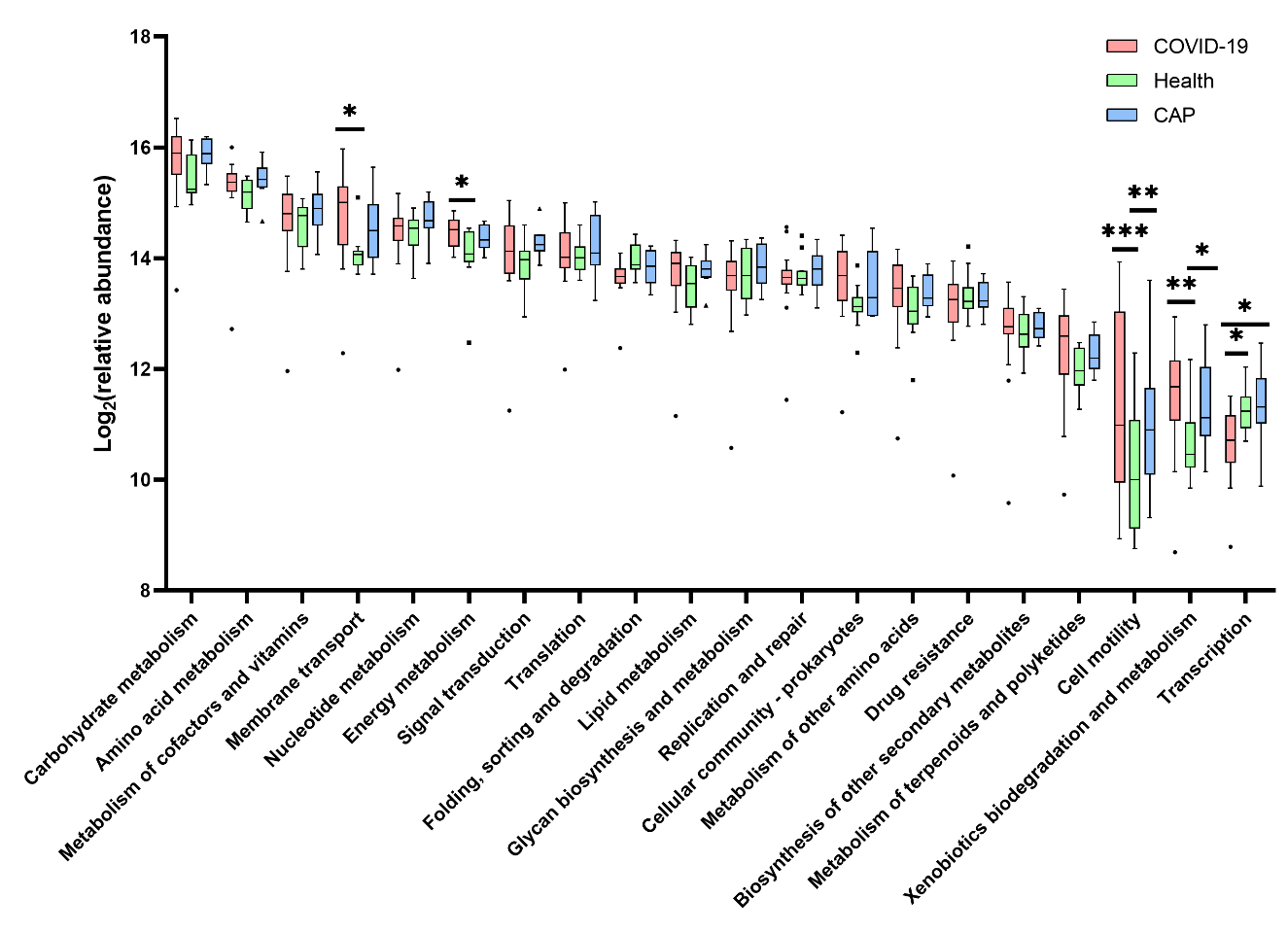


**Supplementary Figure 7** KEGG Level 2 annotation statistics between COVID-19, Health and CAP groups. Significance was marked as **p* < 0.05, ***p* < 0.01, ****p* < 0.001.


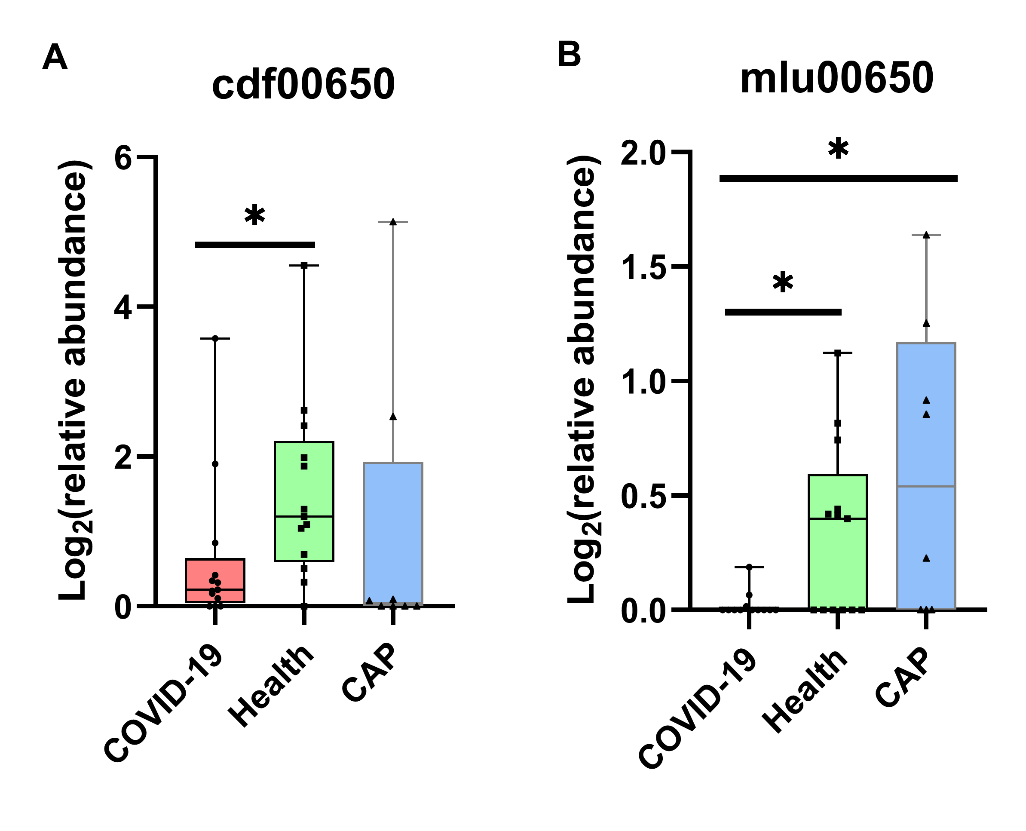


**Supplementary Figure 8** Butyrate synthesis pathway based on KEGG pathway entry annotation. The enzymes involved in cdf00650 and mlu00650 pathway are butyrate kinase and butyryl-CoA: acetate CoA transferase, respectively. Significance was marked as **p* < 0.05, ***p* < 0.01, ****p* < 0.001.


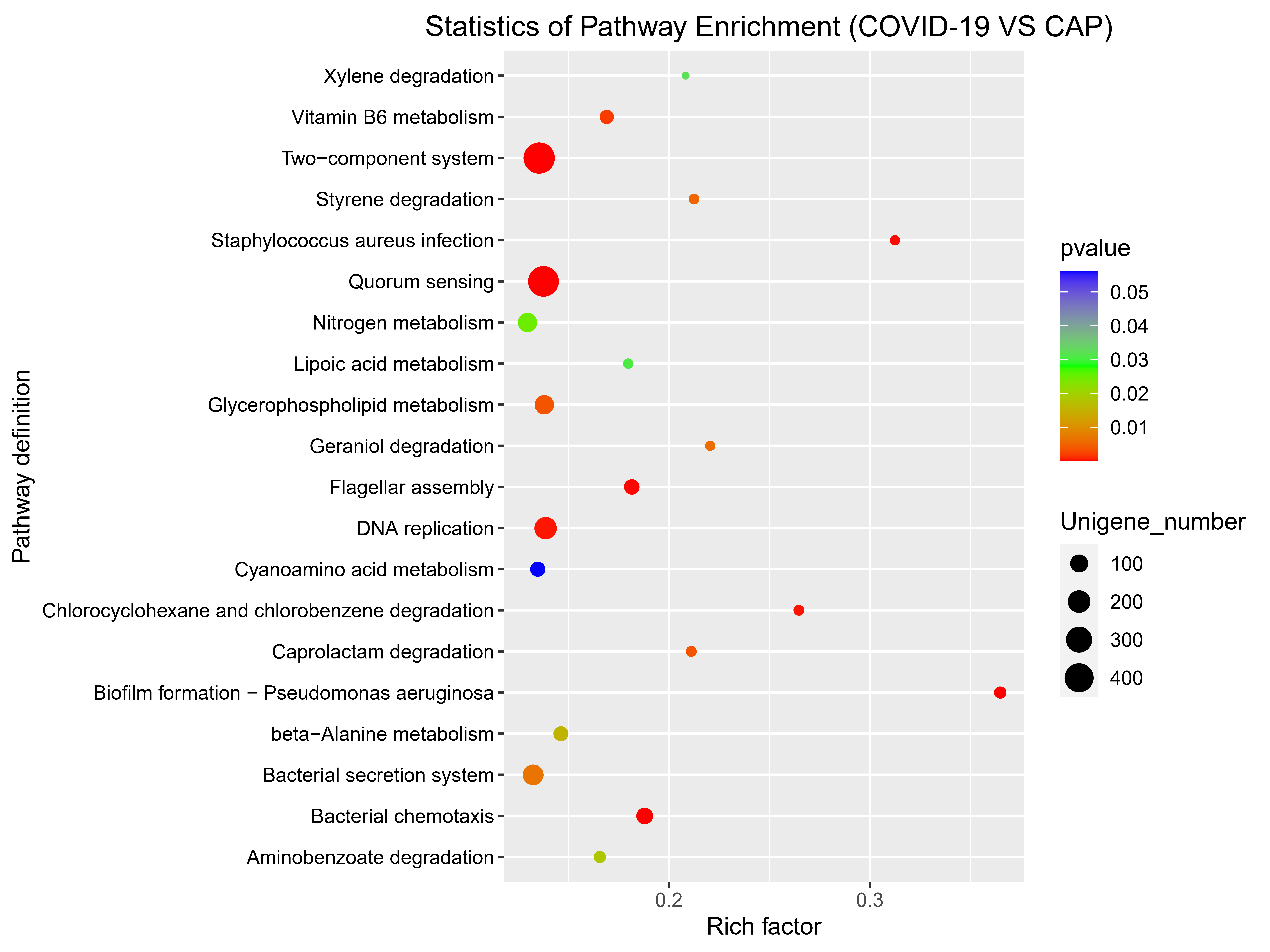


**Supplementary Figure 9** KEGG annotation statistics of different genes between COVID-19 group and CAP group. The size of each circle represents the number of significant unigenes enriched in the corresponding pathway (The significant threshold of differential genes was absolute value of log2 (fold change) ≥1, *p* <0.05). The enrichment factor was calculated using the number of enriched gene divided by the total number of background genes in the corresponding pathway. A pathway with a *p* value <0.03 is considered to be significantly over-represented.


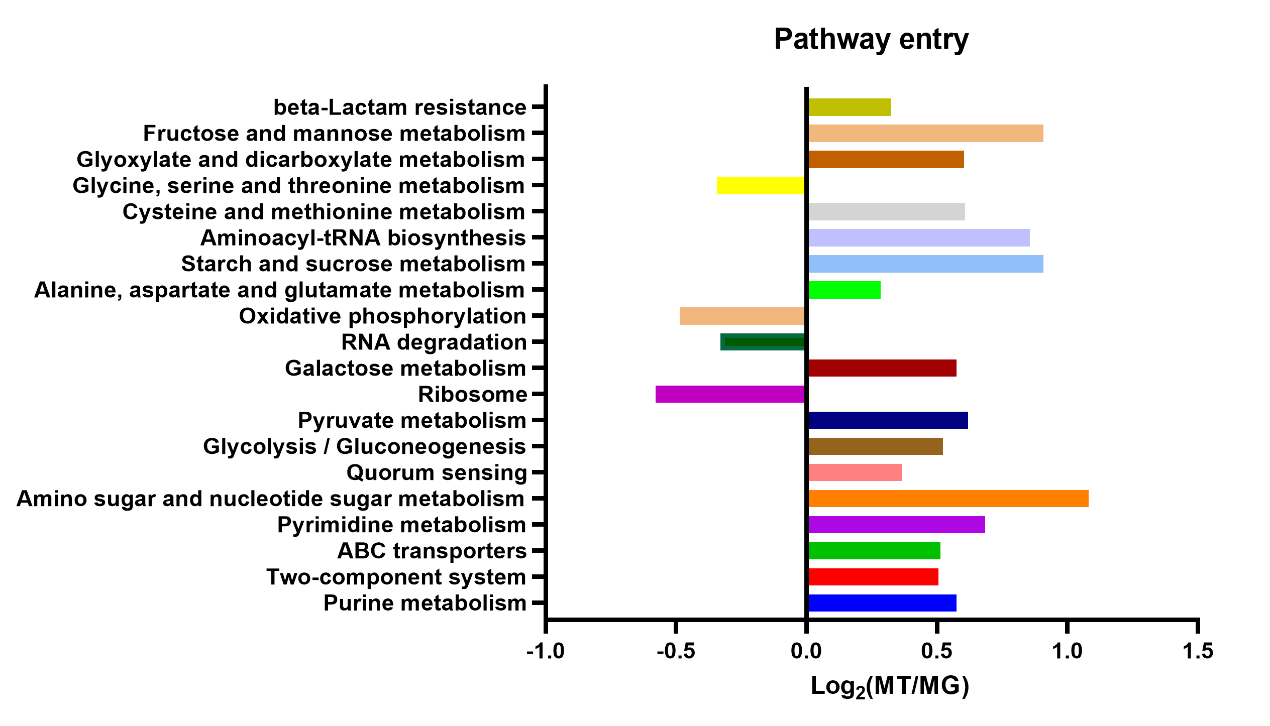


**Supplementary Figure 10** Ratio of mean relative abundance of KEGG pathway entry annotations in MG to that in MT (MT/MG). Log2 is used to data normalization.


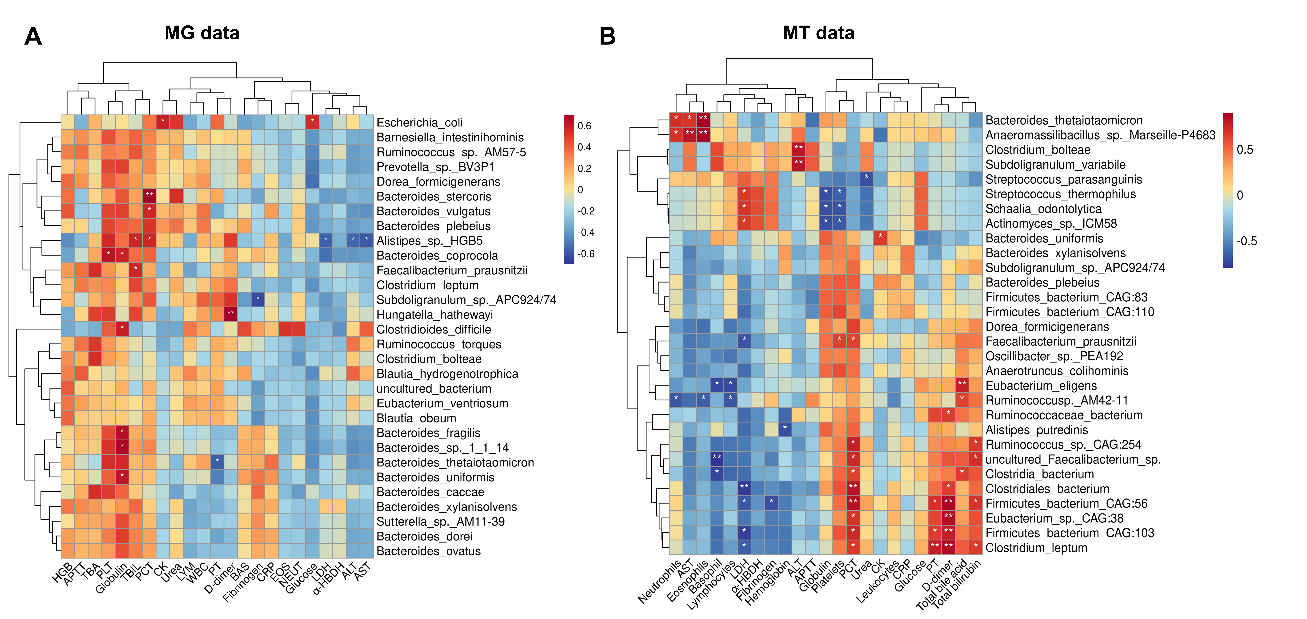


**Supplementary Figure 11** Relationship between clinical information of COVID-19 and species composition (abundance >0.1%) based on metagenome (A) and metatranscriptome (B) data, evaluated by Pearson test. The degree of correlation is indicated by a color gradient from red (positive correlation) to blue (negative correlation). Significance was marked as **p* < 0.05, ***p* < 0.01, ****p* < 0.001.

**Supplementary Table 1** Clinical characteristics of n = 13 healthy controls

| **Characteristics** | Health1* | Health2* | Health3* | Health4  * | Health5* | Health6* | Health7* | Health8* | Health9* | Health10* | Health11* | Health12* | Health13* |
| --- | --- | --- | --- | --- | --- | --- | --- | --- | --- | --- | --- | --- | --- |
| Age（year） | 50.00 | 40.00 | 24.00 | 52.00 | 41.00 | 26.00 | 57.00 | 24.00 | 23.00 | 22.00 | 26.00 | 19.00 | 23.00 |
| Sex | Female | Female | Male | Male | Male | Male | Male | Male | Female | Male | Male | Male | Male |
| BMI | 22.51 | 20.23 | 23.41 | 20.42 | 22.13 | 20.26 | 23.72 | 19.88 | 21.65 | 19.94 | 20.16 | 21.22 | 20.01 |
| **Blood routine** |  | | | | | | | | | | | | |
| Leukocytes (×10^9^ L^-1^; normal range 5.0–12.0) | 5.58 | 5.76 | 7.37 | 11.06 | 4.02 | 7.08 | 6.10 | 6.12 | 6.71 | 7.62 | 5.86 | 9.73 | 10.12 |
| Neutrophils (×10^9^ L^-1^; normal range 2.0–7.2) | 3.19 | 3.54 | 5.13 | 6.76 | 1.93 | 4.16 | 4.04 | 3.59 | 4.49 | 7.12 | 5.34 | 2.81 | 4.64 |
| Lymphocytes (×10^9^ L^-1^; normal range 1.55–4.80) | 1.86 | 1.56 | 3.08 | 2.17 | 2.03 | 2.46 | 1.53 | 2.15 | 2.11 | 4.02 | 3.11 | 2.71 | 1.68 |
| Platelets (×10^9^ L^-1^; normal range 140–440) | 205.00 | 276.00 | 232.00 | 312.00 | 312.00 | 270.00 | 234.00 | 227.00 | 258.50 | 271.00 | 148.00 | 352.00 | 265.00 |
| Eosinophils (×10^9^ L^-1^; normal range 0-0.7) | 0.07 | 0.15 | 0.03 | 0.54 | 0.08 | 0.09 | 0.05 | 0.09 | 0.54 | 0.14 | 0.05 | 0.16 | 0.24 |
| Basophil (×10^9^ L^-1^; normal range 0~0.2) | 0.00 | 0.01 | 0.02 | 0.01 | 0.02 | 0.01 | 0.01 | 0.01 | 0.01 | 0.02 | 0.00 | 0.01 | 0.01 |
| Hemoglobin (g L^-1^; normal range 105–145) | 142.00 | 128.00 | 106.00 | 136.00 | 142.00 | 117.00 | 133.00 | 163.00 | 143.38 | 127.00 | 124.00 | 106.00 | 133.00 |
| **Coagulation function** |  | | | | | | | | | | | | |
| APTT (s; normal range 28–45) | 30.00 | 33.00 | 28.00 | 40.00 | 43.00 | 42.00 | 40.00 | 41.00 | 29.00 | 42.00 | 43.00 | 45.00 | 46.00 |
| PT (s; normal range 11–15) | 13.50 | 12.30 | 14.60 | 12.80 | 15.20 | 12.10 | 14.70 | 12.90 | 11.90 | 13.80 | 12.50 | 14.50 | 13.50 |
| D-dimer (μg^-1^; normal range <0.5) | 0.13 | 0.02 | 0.01 | 0.12 | 0.22 | 0.06 | 0.25 | 0.25 | 0.25 | 0.23 | 0.38 | 0.36 | 0.07 |
| Fibrinogen (g L^-1^; normal range 2.0–4.0) | 2.22 | 2.82 | 3.55 | 2.63 | 3.57 | 3.42 | 2.71 | 3.24 | 2.68 | 3.68 | 3.98 | 2.44 | 2.55 |
| **Blood biochemistry** |  | | | | | | | | | | | | |
| Globulin (g L^-1^; normal range 20.0–30.0) | 22.70 | 27.80 | 31.20 | 22.30 | 27.90 | 29.10 | 27.30 | 26.10 | 27.80 | 21.20 | 23.20 | 28.50 | 20.10 |
| AST (U L^-1^; normal range 5.0–60.0) | 20.00 | 18 | 22.00 | 15.00 | 21.00 | 15.00 | 23.00 | 19.00 | 19.29 | 18.20 | 16.00 | 17.00 | 22.00 |
| ALT (U L^-1^; normal range 9.0–50.0) | 8.00 | 10 | 19.00 | 7.00 | 14.00 | 19.00 | 15.00 | 24.00 | 9.00 | 10.00 | 26.00 | 15.00 | 8.00 |
| Total bilirubin (μmol L^-1^; normal range 2.0–17.0) | 10.60 | 11.90 | 10.40 | 16.00 | 15.50 | 9.30 | 15.70 | 14.80 | 15.03 | 11.60 | 11.10 | 9.80 | 10.60 |
| Total bile acid (μmol L^-1^; normal range 0.1–10) | 1.20 | 2.30 | 1.50 | 2.60 | 0.50 | 5.50 | 2.10 | 3.80 | 6.80 | 8.30 | 5.60 | 3.80 | 4.90 |
| Urea (μmol L^-1^; normal range 2.0–7.0) | 4.68 | 6.36 | 4.12 | 3.97 | 3.55 | 6.45 | 4.71 | 4.97 | 4.85 | 2.51 | 2.39 | 3.15 | 4.88 |
| CK (U L^-1^; normal range 45–390) | 85.00 | 93.00 | 143.00 | 198.70 | 88.00 | 86.00 | 100.00 | 68.00 | 67.90 | 81.50 | 198.00 | 113.00 | 95.00 |
| LDH (U L^-1^; normal range 159–322) | 166.00 | 212.00 | 245.00 | 208.00 | 165.00 | 206.00 | 180.00 | 212.88 | 203.80 | 186.00 | 159.00 | 214.00 | 182.00 |
| α-HBDH (U L^-1^; normal range 90–220) | 100.00 | 215.00 | 92.00 | 145.00 | 116.00 | 175.00 | 169.00 | 145.00 | 164.00 | 153.00 | 185.00 | 167.00 | 155.00 |
| Glucose (mmol L^-1^; normal range 4.1–5.9) | 5.35 | 5.49 | 5.43 | 4.42 | 4.13 | 5.30 | 4.64 | 5.32 | 5.89 | 4.61 | 4.49 | 5.65 | 5.13 |
| **Infection-related biomarkers** |  | | | | | | | | | | | | |
| PCT (ng L^-1^; normal range <0.1) | 0.01 | 0.03 | 0.03 | 0.05 | 0.03 | 0.03 | 0.05 | 0.01 | 0.06 | 0.05 | 0.02 | 0.04 | 0.04 |
| CRP (mg L^-1^; normal range 0.0–6.0) | 2.18 | 1.88 | 1.53 | 5.62 | 3.56 | 2.28 | 1.96 | 0.53 | 2.50 | 3.55 | 1.20 | 0.89 | 1.36 |

# APTT, activated partial thromboplastin time; PT, prothrombin time; AST, aspartate aminotransferase; ALT, alanine aminotransferase; CK, creatine kinase; LDH, lactate dehydrogenase; α-HBDH, α-hydroxybutyric dehydrogenase; CRP, C-reactive protein; PCT, procalcitonin; NA, not available; *Stool sample collection.

**Supplementary Table 2** Clinical characteristics of n = 13 independent patients confirmed with SARS-CoV-2 infection

| **Characteristics** | Patient1* | Patient2* | Patient3* | Patient4* | Patient5* | Patient6* | Patient7* | Patient8* | Patient9* | Patient  10* | Patient  11* | Patient12* | Patient  13* |
| --- | --- | --- | --- | --- | --- | --- | --- | --- | --- | --- | --- | --- | --- |
| Age（year） | 50 | 37 | 24 | 52 | 41 | 26 | 57 | 22 | 24 | 20 | 24 | 21 | 23 |
| Sex | Female | Female | Male | Male | Male | Male | Male | Male | Female | Male | Male | Male | Male |
| BMI | 22.8 | 20.6 | 23.5 | 20.22 | 21.98 | 20.32 | 24.2 | 19.53 | 20.84 | 20.25 | 20.55 | 21.51 | 20.65 |
| **Signs and symptoms at admission** |  | | | | | | | | | | | | |
| Fever | √ | ✘ | ✘ | ✘ | √ | √ | √ | ✘ | ✘ | √ | ✘ | ✘ | ✘ |
| Cough | √ | ✘ | ✘ | √ | √ | ✘ | √ | ✘ | ✘ | √ | ✘ | ✘ | ✘ |
| Sore throat | ✘ | √ | √ | ✘ | ✘ | √ | ✘ | ✘ | ✘ | ✘ | ✘ | ✘ | ✘ |
| Chest distress | ✘ | √ | ✘ | ✘ | ✘ | ✘ | ✘ | ✘ | ✘ | ✘ | ✘ | ✘ | ✘ |
| Diarrhea | ✘ | ✘ | ✘ | ✘ | √ | ✘ | ✘ | ✘ | ✘ | ✘ | ✘ | ✘ | ✘ |
| Degree of severity | Medium | Mild | Mild | Medium | Medium | Medium | Severe | Mild | Mild | Mild | Mild | Mild | Mild |
| **Chest computed tomography scan** |  | | | | | | | | | | | | |
| Lung markings increased | ✘ | √ | √ | ✘ | ✘ | √ | ✘ | ✘ | √ | √ | ✘ | ✘ | √ |
| Mottling and ground-glass opacity | √ | ✘ | ✘ | √ | √ | √ | √ | ✘ | ✘ | ✘ | ✘ | ✘ | ✘ |
| **Blood routine** |  | | | | | | | | | | | | |
| Leukocytes (×10^9^ L^-1^; normal range 5.0–12.0) | 5.93 | 6.2 | 4.44 | 5.55 | 4.08 | 5.64 | 4.81 | 6.29 | 6.59 | 6 | 8.34 | 5.68 | 5.5 |
| Neutrophils (×10^9^ L^-1^; normal range 2.0–7.2) | 6.19 | 3.73 | 3.05 | 3.21 | 2.38 | 3.1 | 3.51 | 4.38 | 4.27 | 3.43 | 5.1 | 3.6 | 3.49 |
| Lymphocytes (×10^9^ L^-1^; normal range 1.55–4.80) | 1.89 | 1.84 | 1.01 | 1.93 | 1.32 | 2.06 | 0.85 | 1.26 | 1.75 | 1.85 | 2.45 | 1.72 | 1.51 |
| Platelets (×10^9^ L^-1^; normal range 140–440) | 249 | 238 | 224 | 121 | 261 | 166 | 141 | 282 | 250 | 204 | 321 | 275 | 201 |
| Eosnophils (×10^9^ L^-1^; normal range 0-0.7) | 2.1 | 0.15 | 0.05 | 0.06 | 0.03 | 0.04 | 0.05 | 0.07 | 0.26 | 0.16 | 0.21 | 0.03 | 0.13 |
| Basophil (×10^9^ L^-1^; normal range 0~0.2) | 0.02 | 0.03 | 0.01 | 0.02 | 0.01 | 0.04 | 0 | 0 | 0.01 | 0 | 0.02 | 0.03 | 0.01 |
| Hemoglobin (g L^-1^; normal range 105–145) | 149 | 165 | 168 | 134 | 149 | 160 | 134 | 120 | 159 | 167 | 158 | 120 | 170 |
| **Coagulation function** |  | | | | | | | | | | | | |
| APTT (s; normal range 28–45) | 21.7 | 34.4 | 31.9 | 29.9 | 32 | 36.4 | 22.66 | 25.7 | 20.9 | 24.32 | 25.7 | 25.3 | 23.2 |
| PT (s; normal range 11–15) | 13.2 | 12.2 | 12.3 | 12.1 | 13.1 | 12.2 | 15.32 | 16.9 | 15.2 | 15 | 11.6 | 12.4 | 10.1 |
| D-dimer (μg^-1^; normal range <0.5) | 0.39 | 0.14 | 0.01 | 0.17 | 0.18 | 0.34 | 0.35 | 2.28 | 0.24 | 0.15 | 0.07 | 0.22 | 0.55 |
| Fibrinogen (g L^-1^; normal range 2.0–4.0) | 2.4 | 2.42 | 2.67 | 3.26 | 2.96 | 2.99 | 1.92 | 1.69 | 1.64 | 1.59 | 2.34 | 2.98 | 2.39 |
| **Blood biochemistry** |  | | | | | | | | | | | | |
| Globulin (g L^-1^; normal range 20.0–30.0) | 27.5 | 26.5 | 27.5 | 21.7 | 26.2 | 26.2 | 24.4 | 25 | 29 | 27 | 29 | 31 | 26 |
| AST (U L^-1^; normal range 5.0–60.0) | 54 | 22 | 17 | 19 | 29 | 44 | 15.3 | 16 | 17 | 21 | 16 | 18 | 19 |
| ALT (U L^-1^; normal range 9.0–50.0) | 62 | 21 | 13 | 13 | 65 | 124 | 25.2 | 12 | 17 | 32 | 15 | 10 | 17 |
| Total bilirubin (μmol L^-1^; normal range 2.0–17.0) | 10.3 | 22.1 | 26.8 | 15.6 | 16.8 | 14.5 | 13.5 | 36.13 | 20.03 | 9.03 | 23.27 | 13.55 | 12.61 |
| Total bile acid (μmol L^-1^; normal range 0.1–10) | 2.5 | 2.3 | 5 | 2.5 | 7.5 | 2.5 | 2.30 | 5.4 | 2.4 | 3.5 | 2.1 | 1.9 | 1.7 |
| Urea (μmol L^-1^; normal range 2.0–7.0) | 3.88 | 4.93 | 4.74 | 2.63 | 3.38 | 5.07 | 5.52 | 5 | 6.3 | 3.6 | 3.4 | 3.3 | 4 |
| CK (U L^-1^; normal range 45–390) | 53 | 127 | 60 | 68 | 81 | 64 | 183 | 67 | 98 | 51 | 101 | 62 | 86 |
| LDH (U L^-1^; normal range 159–322) | 199 | 228 | 218 | 297.93 | 195 | 244.54 | 206 | 142 | 143 | 175 | 172 | 159 | 234 |
| α-HBDH (U L^-1^; normal range 90–220) | 126 | 165 | 157 | 201 | 147 | 160 | 181.5 | 119 | 111 | 135 | 127 | 126 | 186 |
| Glucose (mmol L^-1^; normal range 4.1–5.9) | 5.35 | 4.51 | 4.92 | 5.93 | 6.14 | 4.74 | 8.25 | 5.4 | 5.07 | 4.86 | 5.08 | 4.73 | 4.67 |
| **Infection-related biomarkers** |  | | | | | | | | | | | | |
| PCT (ng L^-1^; normal range <0.1) | 0.02 | 0.041 | 0.049 | 0.02 | 0.021 | 0.02 | 0.043 | 0.08 | 0.1 | 0.05 | 0.05 | 0.05 | 0.05 |
| CRP (mg L^-1^; normal range 0.0–6.0) | 2.72 | 0.23 | 3.53 | 2.41 | 1.01 | 1.44 | 0.87 | 0.2 | 0.56 | 1.17 | 11.88 | 1.13 | 0.5 |
| **Antibiotic therapy at presentation** |  | | | | | | | | | | | | |
| Ceftriaxone | ✘ | ✘ | ✘ | √ | √ | ✘ | ✘ | ✘ | ✘ | √ | ✘ | ✘ | ✘ |
| Moxifloxacin | √ | √ | √ | √ | √ | √ | √ | ✘ | ✘ | ✘ | ✘ | ✘ | ✘ |
| Levofloxacin | ✘ | ✘ | ✘ | ✘ | √ | ✘ | ✘ | ✘ | ✘ | ✘ | ✘ | ✘ | ✘ |
| **Antiviral therapy** |  | | | | | | | | | | | | |
| Oseltamivir | ✘ | ✘ | ✘ | √ | ✘ | √ | ✘ | ✘ | ✘ | ✘ | ✘ | ✘ | ✘ |
| Interferon alpha | √ | √ | √ | √ | √ | ✘ | √ | √ | √ | √ | √ | √ | √ |
| Kaletra | √ | √ | √ | √ | √ | ✘ | √ | √ | √ | √ | √ | √ | √ |
| Ribavirin | ✘ | ✘ | ✘ | ✘ | ✘ | ✘ | √ | ✘ | ✘ | ✘ | ✘ | ✘ | ✘ |

#✓, with; ✗, without; APTT, activated partial thromboplastin time; PT, prothrombin time; AST, aspartate aminotransferase; ALT, alanine aminotransferase; CK, creatine kinase; LDH, lactate dehydrogenase; α-HBDH, α-hydroxybutyric dehydrogenase; CRP, C-reactive protein; PCT, procalcitonin; NA, not available; *Stool sample collection.

**Supplementary Table 3.1** Clinical characteristics of n = 24 independent patients with community-acquired pneumonia (1-13 cases)

| **Characteristics** | Patient1 | Patient2* | Patient3 | Patient4 | Patient5 | Patient6 | Patient7 | Patient8 | Patient9 | Patient10 | Patient11* | Patient12* | Patient13 |
| --- | --- | --- | --- | --- | --- | --- | --- | --- | --- | --- | --- | --- | --- |
| Age（year） | 25 | 54 | 25 | 50 | 30 | 35 | 28 | 39 | 36 | 38 | 27 | 29 | 57 |
| Sex | Female | Male | Female | Female | Male | Male | Male | Male | Female | Female | Female | Female | Female |
| BMI |  | 23.62 |  |  |  |  |  |  |  |  | 23.55 | 22.26 |  |
| **Signs and symptoms at admission** |  |  |  |  |  |  |  |  |  |  |  |  |  |
| Fever | √ | √ | √ | √ | √ | √ | √ | ✘ | √ | √ | √ | √ | √ |
| Cough | √ | ✘ | √ | √ | √ | √ | √ | √ | √ | √ | √ | √ | ✘ |
| Sore throat | √ | ✘ | ✘ | ✘ | ✘ | ✘ | √ | ✘ | ✘ | ✘ | ✘ | ✘ | √ |
| Chest distress | ✘ | ✘ | ✘ | ✘ | ✘ | ✘ | ✘ | ✘ | ✘ | ✘ | ✘ | ✘ | ✘ |
| Diarrhea | ✘ | ✘ | ✘ | ✘ | ✘ | ✘ | ✘ | ✘ | ✘ | ✘ | ✘ | ✘ | ✘ |
| More than one sign or symptom | √ | ✘ | √ | √ | √ | √ | √ | ✘ | √ | √ | √ | √ | √ |
| Chest computed tomography scan |  |  |  |  |  |  |  |  |  |  |  |  |  |
| Lung markings increased | √ | ✘ | ✘ | ✘ | ✘ | ✘ | ✘ | ✘ | ✘ | ✘ | ✘ | ✘ | √ |
| Mottling and ground-glass opacity | ✘ | √ | √ | √ | √ | √ | √ | √ | √ | √ | √ | √ | ✘ |
| **Blood routine** |  |  |  |  |  |  |  |  |  |  |  |  |  |
| Leukocytes (× 10^9^ L^-1^; normal range 5.0–12.0) | 4.98 | 6.59 | 10.43 | 6.35 | 6.4 | 6.15 | 6.69 | 6.23 | 3.19 | 7.21 | 4.06 | 4.14 | 5.88 |
| Neutrophils (× 10^9^ L^-1^; normal range 2.0–7.2) | 3.15 | 3.48 | 6.95 | 4.6 | 4.09 | 3.03 | 4.12 | 4.4 | 1.94 | 3.91 | 2.13 | 2.6 | 4.24 |
| Lymphocytes (×10^9^ L^-1^; normal range 1.55–4.80) | 1.46 | 2.2 | 2.83 | 1.39 | 1.84 | 2.57 | 1.98 | 1.42 | 1.08 | 2.65 | 1.56 | 1.27 | 1.13 |
| Platelets (×10^9^ L^-1^; normal range 140–440) | 208 | 242 | 338 | 176 | 232 | 259 | 181 | 268 | 203 | 281 | 479 | 217 | 204 |
| Eosinophils (× 10^9^ L^-1^; normal range 0.005-0.05) | 0.03 | 0.46 | 0.1 | 0.06 | 0.16 | 0.17 | 0.22 | 0.11 | 0.18 | 0.24 | 0.04 | 0.04 | 0.09 |
| Basophil (× 10^9^ L^-1^; normal range 0~0.0075) | 0.04 | 0.03 | 0.02 | 0.02 | 0.02 | 0.01 | 0.01 | 0.01 | 0.01 | 0.04 | 0 | 0 | 0.1 |
| Hemoglobin (g L^-1^; normal range 105–145) | 124 | 152 | 140 | 156 | 130 | 169 | 178 | 166 | 119 | 136 | 142 | 145 | 130 |
| **Coagulation function** |  |  |  |  |  |  |  |  |  |  |  |  |  |
| APTT (s; normal range 28–45) | 29.5 | 36.5 | 31.5 | 31.2 | 35.2 | 30.3 | 29.6 | 31.4 | 30.9 | 30.8 | 33.9 | 34.1 | 34.5 |
| PT (s; normal range 11–15) | 14.4 | 14.7 | 12.9 | 12.9 | 13.5 | 13 | 13.1 | 11.3 | 13.1 | 11.9 | 12.1 | 13 | 14.73 |
| D-dimer (μg^-1^; normal range <0.5) | 0.72 | 0.39 | 0.23 | 0.59 | 0.25 | 1.13 | 0.77 | 0.48 | 0.43 | 0.41 | 16.79 | 0.23 | 1.86 |
| Fibrinogen (g L^-1^; normal range 2.0–4.0) | 4.1 | 4.52 | 5.19 | 4.95 | 3.06 | 4.27 | 3.89 | 3.6 | 3.56 | 4.52 | 4.59 | 3.84 | 3.87 |
| **Blood biochemistry** |  |  |  |  |  |  |  |  |  |  |  |  |  |
| γ－Globulin (g L^−1^; normal range 20.0–30.0) | 26.6 | 30.3 | 30.8 | 36.7 | 27.9 | 25.6 | 26.9 | 21.4 | 25.8 | 33.2 | 25.1 | 30.2 | 34.6 |
| AST (U L^-1^; normal range 5.0–60.0) | 14 | 47 | 21 | 27 | 24 | 43 | 45.1 | 17 | 19 | 18 | 185 | 20 | 17 |
| ALT (U L^-1^; normal range 9.0–50.0) | 7 | 65 | 22 | 25 | 11 | 76 | 43.4 | 28 | 14 | 17 | 178 | 10 | 23.1 |
| Total bilirubin (μmol L^-1^; normal range 2.0–17.0) | 15.3 | 11.1 | 5.7 | 12.2 | 5.6 | 17.5 | 17.5 | 20.8 | 8.1 | 13.1 | 5.5 | 18.4 | 13.8 |
| Total bile acid (μmol L^-1^; normal range 0.1–10) | 2.9 | 1.4 | 3.3 | 13.2 | 12.1 | 2.5 | 8.1 | 16.2 | 1.5 | 1 | 1.8 | 1.6 | 0.3 |
| Urea (μmol L^-1^; normal range 2.0–7.0) | 2.15 | 4.98 | 4.18 | 2.93 | 2.46 | 5.42 | 3.21 | 5.37 | 3.88 | 3.18 | 2.9 | 4.41 | 3.35 |
| CK (U L^-1^; normal range 45–390) | 36 | 208 | 82 | 83 | 153 | 595 | 128.7 | 77 | 62 | 96 | 98 | 63 | 72.9 |
| LDH (U L-1; normal range 159–322) | 148 | 325.9 | 151 | 216 | 232 | 239.25 | 238 | 215 | 201.88 | 179.44 | 612.88 | 233.69 | 200 |
| α-HBDH (U L-1; normal range 90–220) | 110 | 212 | 114 | 154 | 161 | 157 | 185.9 | 154 | 146 | 135 | 319 | 158 | 176 |
| Glucose (mmol L^-1^; normal range 4.1–5.9) | 8.9 | 5.53 | 6.39 | 5.94 | 10.83 | 4.24 | 4.3 | 5.03 | 4.46 | 5.23 | 6.03 | 4.16 | 5.94 |
| **infection-related biomarkers** |  |  |  |  |  |  |  |  |  |  |  |  |  |
| PCT (ng L^-1^; normal range <0.1) | 0.031 | 0.1 | 0.058 | 0.08 | 0.029 | 0.079 | 0.033 | <0.02 | 0.029 | 0.04 | 0.083 | <0.02 | 0.06 |
| CRP (mg L^-1^; normal range 0.0–6.0) | 1.18 | 13.07 | 1.88 | 4.03 | 1.13 | 4.62 | 0.56 | 0.28 | 11.76 | 0.96 | 22.2 | 0.83 | 72.2 |
| **Antibiotic therapy at presentation** |  |  |  |  |  |  |  |  |  |  |  |  |  |
| Ceftriaxone | ✘ | ✘ | ✘ | ✘ | ✘ | ✘ | ✘ | ✘ | ✘ | ✘ | √ | ✘ | ✘ |
| Moxifloxacin | √ | √ | √ | √ | √ | √ | √ | √ | √ | √ | ✘ | √ | √ |
| Levofloxacin | ✘ | ✘ | ✘ | ✘ | ✘ | ✘ | ✘ | ✘ | ✘ | ✘ | ✘ | ✘ | ✘ |
| **Antiviral therapy** |  |  |  |  |  |  |  |  |  |  |  |  |  |
| Oseltamivir | ✘ | √ | ✘ | ✘ | ✘ | ✘ | ✘ | ✘ | ✘ | ✘ | √ | √ | ✘ |
| Interferon | ✘ | ✘ | ✘ | ✘ | ✘ | ✘ | ✘ | ✘ | √ | √ | √ | √ | ✘ |
| Kaletra | ✘ | ✘ | ✘ | ✘ | ✘ | ✘ | ✘ | ✘ | ✘ | ✘ | ✘ | ✘ | ✘ |
| Ribavirin | ✘ | ✘ | ✘ | ✘ | ✘ | ✘ | √ | ✘ | ✘ | √ | ✘ | ✘ | ✘ |

#✓, with; ✗, without; APTT, activated partial thromboplastin time; PT, prothrombin time; AST, aspartate aminotransferase; ALT, alanine aminotransferase; CK, creatine kinase; LDH, lactate dehydrogenase; α-HBDH, α-hydroxybutyric dehydrogenase; C-reactive protein; PCT, procalcitonin; NA, not available; *Stool sample collection.

**Supplementary Table 3.2** Clinical characteristics of n = 24 independent patients with community-acquired pneumonia (14-24 cases)

| **Characteristics** | Patient14 | Patient15* | Patient16* | Patient17* | Patient18 | Patient19* | Patient20 | Patient21 | Patient22 | Patient23* | Patient24 |
| --- | --- | --- | --- | --- | --- | --- | --- | --- | --- | --- | --- |
| Age（year） | 29 | 30 | 31 | 49 | 58 | 41 | 33 | 39 | 30 | 31 | 27 |
| sex | Male | Female | Female | Male | Male | Male | Female | Male | Male | Female | Female |
| BMI |  | 23.15 | 20.33 | 21.42 |  | 20.54 |  |  |  | 21.82 |  |
| **Signs and symptoms at admission** |  |  |  |  |  |  |  |  |  |  |  |
| Fever | √ | √ | √ | √ | √ | √ | √ | √ | √ | √ | √ |
| Cough | ✘ | ✘ | ✘ | ✘ | √ | ✘ | ✘ | √ | √ | ✘ | √ |
| Sore throat | ✘ | ✘ | √ | ✘ | ✘ | ✘ | √ | ✘ | ✘ | ✘ | ✘ |
| Chest distress | ✘ | ✘ | ✘ | ✘ | ✘ | ✘ | ✘ | ✘ | ✘ | ✘ | ✘ |
| Diarrhea | ✘ | ✘ | ✘ | ✘ | ✘ | ✘ | ✘ | ✘ | ✘ | ✘ | ✘ |
| More than one sign or symptom | ✘ | ✘ | √ | ✘ | √ | ✘ | √ | √ | √ | ✘ | √ |
| Chest computed tomography scan |  |  |  |  |  |  |  |  |  |  |  |
| Lung markings increased | ✘ | √ | ✘ | √ | ✘ | ✘ | ✘ | ✘ | ✘ | √ | ✘ |
| Mottling and ground-glass opacity | √ | ✘ | √ | ✘ | √ | √ | √ | √ | √ | ✘ | √ |
| **Blood routine** |  |  |  |  |  |  |  |  |  |  |  |
| Leukocytes (× 10^9^ L^-1^; normal range 5.0–12.0) | 8.74 | 5.35 | 6.1 | 9.13 | 5.74 | 5.99 | 5.76 | 7.47 | 7.76 | 5.22 | 6.9 |
| Neutrophils (× 10^9^ L^-1^; normal range 2.0–7.2) | 5.71 | 2.72 | 4.07 | 6.88 | 3.54 | 3.52 | 3.02 | 4.21 | 6.64 | 1.59 | 4.52 |
| Lymphocytes (×10^9^ L^-1^; normal range 1.55–4.80) | 2.44 | 2.23 | 1.57 | 1.73 | 1.82 | 1.81 | 2.31 | 2.65 | 0.78 | 1.3 | 1.67 |
| Platelets (×10^9^ L^-1^; normal range 140–440) | 386 | 136 | 354 | 268 | 197 | 259 | 339 | 148 | 139 | 171 | 346 |
| Eosinophils (× 10^9^ L^-1^; normal range 0.005-0.05) | 0.26 | 0.09 | 0.09 | 0.11 | 0.03 | 0.38 | 0.09 | 0.12 | 0.01 | 0.13 | 0.27 |
| Basophil (× 10^9^ L^-1^; normal range 0~0.0075) | 0 | 0.03 | 0.01 | 0 | 0.01 | 0.03 | 0.03 | 0.03 | 0.01 | 0 | 0.03 |
| Hemoglobin (g L^-1^; normal range 105–145) | 185 | 123 | 124 | 151 | 153 | 162 | 148 | 151 | 155 | 145 | 138 |
| **Coagulation function** |  |  |  |  |  |  |  |  |  |  |  |
| APTT (s; normal range 28–45) | 29.82 | 44.63 | 35.5 | 30.63 | 37.46 | 31.01 | 32.91 | 35.12 | 32.08 | 31.55 | 21.36 |
| PT (s; normal range 11–15) | 14.39 | 12.67 | 13.38 | 11.77 | 15.69 | 16.09 | 14.56 | 14.48 | 16.09 | 12.3 | 15.17 |
| D-dimer (μg^-1^; normal range <0.5) | 0.14 | 0.56 | 1.68 | 0.37 | 0.44 | 0.32 | 0.15 | 1.62 | 0.35 | 0.22 | 0.18 |
| Fibrinogen (g L^-1^; normal range 2.0–4.0) | 3.35 | 1.87 | 4.05 | 2.82 | 2.947 | 2.86 | 2.63 | 2.91 | 2.4 | 3.5 | 2.68 |
| **Blood biochemistry** |  |  |  |  |  |  |  |  |  |  |  |
| γ－Globulin (g L^−1^; normal range 20.0–30.0) | 26.4 | 21.7 | 32.6 | 50.7 | 28.5 | 28.2 | 30 | 30 | 27.2 | 34.8 | 31.2 |
| AST (U L^-1^; normal range 5.0–60.0) | 26.6 | 14 | 18.7 | 22.3 | 32.7 | 13.7 | 15 | 20.6 | 12.8 | 20.1 | 20.4 |
| ALT (U L^-1^; normal range 9.0–50.0) | 47.2 | 5.6 | 12.8 | 16.9 | 42.9 | 9.9 | 13.9 | 24.7 | 11.3 | 11 | 18.2 |
| Total bilirubin (μmol L^-1^; normal range 2.0–17.0) | 14.8 | 9.8 | 7.9 | 7.9 | 16.9 | 9.7 | 8.3 | 9 | 20.8 | 24.3 | 7.5 |
| Total bile acid (μmol L^-1^; normal range 0.1–10) | 0.5 | 1.7 | 8.2 | 6 | 7 | 0.3 | 0 | 0 | 2.5 | 1.7 | 0.9 |
| Urea (μmol L^-1^; normal range 2.0–7.0) | 3.28 | 3.79 | 2.6 | 4.25 | 6.06 | 2.11 | 3.81 | 3.74 | 4.41 | 5.52 | 3.81 |
| CK (U L^-1^; normal range 45–390) | 91.5 | 40.3 | 109.2 | 133.9 | 186.9 | 53.1 | 37.6 | 66.4 | 92.9 | 52.9 | 64.4 |
| LDH (U L-1; normal range 159–322) | 177 | 118 | 149 | 214.00 | 193 | 186 | 105.83 | 177.2 | 136 | 166 | 322 |
| α-HBDH (U L-1; normal range 90–220) | 155.4 | 107.5 | 131.8 | 157.00 | 141.9 | 162.3 | 83 | 148.1 | 116.5 | 145.4 | 237.3 |
| Glucose (mmol L^-1^; normal range 4.1–5.9) | 5.65 | 4.04 | 5.34 | 8.85 | 5.31 | 5.64 | 4.64 | 5.74 | 5.83 | 4.91 | 5.2 |
| **infection-related biomarkers** |  |  |  |  |  |  |  |  |  |  |  |
| PCT (ng L^-1^; normal range <0.1) | 0.05 | 0.07 | 0.15 | 0.04 | 0.03 | 0.03 | 0.06 | 0.05 | 0.03 | 0.11 | NA |
| CRP (mg L^-1^; normal range 0.0–6.0) | 78.17 | 0.5 | 109.95 | 1.59 | 19.41 | 15.09 | 0.54 | 15.75 | 25.05 | 2.22 | 4.5 |
| **Antibiotic therapy at presentation** |  |  |  |  |  |  |  |  |  |  |  |
| Ceftriaxone | ✘ | √ | ✘ | ✘ | ✘ | ✘ | ✘ | ✘ | ✘ | ✘ | √ |
| Moxifloxacin | √ | ✘ | ✘ | √ | √ | √ | √ | ✘ | √ | √ | √ |
| Levofloxacin | ✘ | ✘ | √ | ✘ | ✘ | ✘ | ✘ | √ | ✘ | ✘ | ✘ |
| **Antiviral therapy** |  |  |  |  |  |  |  |  |  |  |  |
| Oseltamivir | ✘ | ✘ | ✘ | ✘ | ✘ | ✘ | ✘ | ✘ | ✘ | √ | √ |
| Interferon | ✘ | ✘ | ✘ | ✘ | ✘ | ✘ | ✘ | ✘ | ✘ | ✘ | ✘ |
| Kaletra | ✘ | ✘ | ✘ | ✘ | ✘ | ✘ | ✘ | ✘ | ✘ | ✘ | ✘ |
| Ribavirin | √ | √ | ✘ | ✘ | ✘ | ✘ | √ | ✘ | ✘ | ✘ | ✘ |

#✓, with; ✗, without; APTT, activated partial thromboplastin time; PT, prothrombin time; AST, aspartate aminotransferase; ALT, alanine aminotransferase; CK, creatine kinase; LDH, lactate dehydrogenase; α-HBDH, α-hydroxybutyric dehydrogenase; CRP, C-reactive protein; PCT, procalcitonin; NA, not available; *Stool sample collection.

**Supplementary Table 4** MG sequence statistics of COVID-19 patients (n=13, 20 samples), healthy controls (n=13, 13 samples) and CAP patients (n=8, 8 samples)

| **Classification** | ***Read*** | | | ***Contig*** | | |
| --- | --- | --- | --- | --- | --- | --- |
|  | Row data (Giga) | Clean data  (Giga) | Q20% | Contig numbers | Assembly length (bp) | N50 (bp) |
| COVID-19.1 (i) | 12.1 | 11.52 | 97.28 | 479179 | 573648764 | 1398 |
| COVID-19.1 (ii) | 12.22 | 11.46 | 97.41 | 121651 | 152180555 | 1350 |
| COVID-19.2 (i) | 10.7 | 10.32 | 97.3 | 84739 | 161275876 | 3312 |
| COVID-19.2 (ii) | 11.72 | 11.19 | 97.41 | 14731 | 27814242 | 3054 |
| COVID-19.3 | 12.34 | 11.79 | 97.39 | 83884 | 107661030 | 1523 |
| COVID-19.4 | 9.76 | 9.31 | 97.17 | 57722 | 77443558 | 1666 |
| COVID-19.5 (i) | 11.22 | 10.78 | 97.27 | 81368 | 214175563 | 7653 |
| COVID-19.5 (ii) | 11.8 | 10.79 | 97.34 | 108564 | 234620048 | 4494 |
| COVID-19.6 | 9.63 | 10.8 | 97.98 | 65537 | 123655285 | 3807 |
| COVID-19.7 | 13.8 | 10.81 | 98.47 | 52491 | 73388562 | 1782 |
| COVID-19.8 | 8.82 | 10.82 | 99.06 | 37211 | 53428998 | 1885 |
| COVID-19.9 (i) | 11.57 | 10.83 | 97.35 | 40207 | 99685300 | 9333 |
| COVID-19.9 (ii) | 12.26 | 10.84 | 97.31 | 12175 | 50765713 | 19821 |
| COVID-19.10 (i) | 25.17 | 10.85 | 98.33 | 53926 | 121300662 | 5426 |
| COVID-19.10 (ii) | 17.16 | 10.86 | 97.57 | 12239 | 39057680 | 10780 |
| COVID-19.10 (iii) | 9.37 | 10.87 | 97.96 | 11297 | 35024615 | 18756 |
| COVID-19.11 (i) | 10.86 | 10.88 | 98.43 | 111339 | 216215450 | 4165 |
| COVID-19.11 (ii) | 10.39 | 10.89 | 97.3 | 85168 | 157053399 | 3889 |
| COVID-19.12 | 10.53 | 10.9 | 97.13 | 101778 | 202645823 | 4845 |
| COVID-19.13 | 8.28 | 10.91 | 98.81 | 110587 | 227005201 | 4510 |
| Healthy control.1 | 9.47 | 10.92 | 97.83 | 103378 | 222638608 | 4533 |
| Healthy control.2 | 8.31 | 10.93 | 98.03 | 79298 | 151938996 | 3559 |
| Healthy control.3 | 8.66 | 10.94 | 98.01 | 64317 | 171709077 | 8675 |
| Healthy control.4 | 7.17 | 10.95 | 98.66 | 77183 | 179513963 | 5941 |
| Healthy control.5 | 11.9 | 10.96 | 97.18 | 142265 | 259112218 | 3038 |
| Healthy control.6 | 13.1 | 10.97 | 98.75 | 118755 | 181808673 | 2062 |
| Healthy control.7 | 14.03 | 10.98 | 97.2 | 143851 | 246165008 | 2690 |
| Healthy control.8 | 10.68 | 10.99 | 98.11 | 73616 | 103674137 | 1757 |
| Healthy control.9 | 10.06 | 10.1 | 98.08 | 65751 | 131863011 | 3926 |
| Healthy control.10 | 19.48 | 10.101 | 98.27 | 153030 | 300947012 | 3875 |
| Healthy control.11 | 11.36 | 10.102 | 98.47 | 43523 | 78546778 | 3613 |
| Healthy control.12 | 10.39 | 10.103 | 97.11 | 101671 | 187298943 | 3450 |
| Healthy control.13 | 11.49 | 10.104 | 97.26 | 66825 | 159221037 | 6113 |
| Pneumonia case.1 | 18.51 | 10.105 | 98.3 | 25464 | 55916559 | 4725 |
| Pneumonia case.2 | 10.26 | 10.106 | 98.4 | 66828 | 157382923 | 6150 |
| Pneumonia case.3 | 18.3 | 10.107 | 98.35 | 41449 | 97833823 | 6600 |
| Pneumonia case.4 | 9 | 10.108 | 98.91 | 49022 | 97459139 | 4083 |
| Pneumonia case.5 | 16.58 | 10.109 | 98 | 22974 | 72947394 | 10323 |
| Pneumonia case.6 | 15.52 | 10.11 | 98.35 | 38669 | 106991755 | 7870 |
| Pneumonia case.7 | 10.1 | 10.111 | 98.01 | 62080 | 139014225 | 5742 |
| Pneumonia case.8 | 8.36 | 10.112 | 97.96 | 141234 | 178151464 | 1452 |

*The Arabic numerals represent the patient number, and the Roman numerals represent the different samples from each patient.

**Supplementary Table 5** MT sequence statistics of COVID-19 patients (n=10, 10 samples)

| **Classification** | ***Read*** | | | ***Contig*** | | |
| --- | --- | --- | --- | --- | --- | --- |
|  | Row data (Giga) | Clean data  (Giga) | Q20% | Contig numbers | Assembly length (bp) | N50 (bp) |
| COVID-19.1 | 17.16 | 16.26 | 97.57 | 554667 | 525101459 | 987 |
| COVID-19.2 | 14.38 | 13.92 | 98.03 | 23272 | 18369520 | 753 |
| COVID-19.3 | 19.48 | 19.14 | 98.27 | 71711 | 102245013 | 1953 |
| COVID-19.4 | 25.17 | 24.27 | 98.33 | 92217 | 106191851 | 1282 |
| COVID-19.5 | 18.51 | 18.23 | 98.3 | 111499 | 247235382 | 3753 |
| COVID-19.6 | 10.06 | 9.85 | 98.08 | 66523 | 137694873 | 3405 |
| COVID-19.8 | 16.61 | 15.67 | 98.4 | 34580 | 46149253 | 1609 |
| COVID-19.11 | 16.58 | 16.23 | 98 | 129566 | 245319879 | 2938 |
| COVID-19.12 | 18.30 | 18.03 | 98.35 | 108742 | 203015035 | 2782 |
| COVID-19.13 | 15.52 | 15.29 | 98.35 | 128822 | 243673530 | 2901 |

*The Arabic numerals represent the patient number.

**Supplementary Table 6** PERMANOVA and ANOSIM test for pairwise comparisons among the Baseline, Last follow up, Health and CAP groups

| **Group** | **ANOSIM**  **(Statistic R)** | ***p* value** | **PERMANOVA**  **(R^2^)** | ***p* value** |
| --- | --- | --- | --- | --- |
| Baseline VS Health | 0.08 | 0.04 | 0.09 | 0.02 |
| Baseline VS CAP | -0.04 | 0.63 | 0.04 | 0.66 |
| Baseline VS Last follow-up | 0.10 | 0.19 | 0.04 | 0.60 |
| Health VS CAP | 0.17 | 0.04 | 0.08 | 0.08 |
| Health VS Last follow-up | 0.51 | 0.00 | 0.22 | 0.00 |
| CAP VS Last follow-up | 0.07 | 0.20 | 0.21 | 0.02 |

**Supplementary Table 7** Gut microbial composition of different cohorts based on MG data

| **Taxon** | **Baseline** | | **Health** | | **Last follow up** | | **CAP** | |
| --- | --- | --- | --- | --- | --- | --- | --- | --- |
| **Phylum** | **Mean (%)** | **SEM** | **Mean (%)** | **SEM** | **Mean (%)** | **SEM** | **Mean (%)** | **SEM** |
| Bacteroidetes | 25.08 | 5.26 | 46.74 | 4.14 | 20.93 | 7.79 | 39.02 | 8.05 |
| Firmicutes | 24.10 | 3.83 | 23.83 | 2.74 | 17.84 | 9.62 | 20.37 | 4.79 |
| Proteobacteria | 12.79 | 4.27 | 7.07 | 2.99 | 47.28 | 14.35 | 16.86 | 10.74 |
| Actinobacteria | 6.93 | 3.28 | 7.35 | 0.62 | 0.81 | 0.32 | 7.04 | 1.15 |
| Verrucomicrobia | 4.15 | 2.81 | 1.31 | 0.33 | 0.02 | 0.01 | 3.38 | 2.31 |
| **Family** | **Mean (%)** | **SEM** | **Mean (%)** | **SEM** | **Mean (%)** | **SEM** | **Mean (%)** | **SEM** |
| Bacteroidaceae | 16.97 | 4.36 | 27.45 | 5.02 | 27.36 | 6.90 | 15.81 | 5.94 |
| Enterobacteriaceae | 10.45 | 3.94 | 4.06 | 2.69 | 14.46 | 9.76 | 41.87 | 12.92 |
| Ruminococcaceae | 9.95 | 1.58 | 9.59 | 1.59 | 8.41 | 2.54 | 12.67 | 8.77 |
| Prevotellaceae | 2.48 | 0.87 | 10.04 | 2.55 | 2.49 | 1.02 | 1.63 | 0.96 |
| Lachnospiraceae | 3.31 | 0.74 | 4.65 | 0.69 | 4.36 | 1.07 | 3.02 | 0.41 |
| Akkermansiaceae | 4.20 | 2.84 | 0.03 | 0.01 | 4.08 | 0.97 | 2.18 | 0.79 |
| Rikenellaceae | 1.96 | 0.80 | 3.22 | 1.40 | 2.23 | 0.57 | 1.19 | 0.53 |
| Streptococcaceae | 3.39 | 1.77 | 2.51 | 1.09 | 5.17 | 3.88 | 0.03 | 0.01 |
| Actinomycetaceae | 4.27 | 3.38 | 0.19 | 0.04 | 3.03 | 1.50 | 0.59 | 0.50 |
| Bifidobacteriaceae | 2.17 | 1.32 | 0.76 | 0.29 | 1.13 | 0.60 | 0.50 | 0.11 |
| Clostridiaceae | 1.72 | 0.32 | 1.80 | 0.24 | 0.42 | 0.24 | 0.26 | 0.09 |
| **Genus** | **Mean (%)** | **SEM** | **Mean (%)** | **SEM** | **Mean (%)** | **SEM** | **Mean (%)** | **SEM** |
| *Bacteroides* | 16.94 | 4.35 | 27.45 | 5.02 | 15.81 | 5.94 | 27.35 | 6.90 |
| *Escherichia* | 7.60 | 3.21 | 3.01 | 2.10 | 33.11 | 10.30 | 11.00 | 7.45 |
| *Prevotella* | 2.38 | 0.87 | 9.87 | 2.56 | 1.60 | 0.96 | 2.45 | 1.02 |
| *Gemmiger* | 2.83 | 0.67 | 1.06 | 0.34 | 7.75 | 6.71 | 1.64 | 0.90 |
| *Akkermansia* | 4.20 | 2.84 | 0.03 | 0.01 | 0.03 | 0.01 | 5.17 | 3.88 |
| *Alistipes* | 1.96 | 0.80 | 3.21 | 1.40 | 0.59 | 0.49 | 3.02 | 1.50 |
| *Faecalibacterium* | 2.27 | 0.78 | 2.80 | 0.80 | 0.67 | 0.35 | 2.98 | 1.84 |
| *Streptococcus* | 3.35 | 1.76 | 2.47 | 1.09 | 0.47 | 0.10 | 1.10 | 0.60 |
| *Subdoligranulum* | 2.24 | 0.51 | 1.98 | 0.73 | 3.08 | 1.81 | 1.61 | 0.55 |
| *Bifidobacterium* | 2.12 | 1.33 | 0.76 | 0.29 | 0.42 | 0.23 | 2.34 | 1.59 |
| *Actinomyces* | 3.19 | 2.53 | 0.12 | 0.03 | 0.12 | 0.07 | 0.23 | 0.18 |
| *Blautia* | 1.02 | 0.24 | 1.59 | 0.26 | 0.41 | 0.19 | 0.79 | 0.19 |
| *Veillonella* | 0.24 | 0.06 | 0.41 | 0.11 | 0.88 | 0.47 | 2.71 | 2.21 |
| *Clostridium* | 1.12 | 0.21 | 1.01 | 0.14 | 0.40 | 0.21 | 0.53 | 0.20 |
| **Species** | **Mean (%)** | **SEM** | **Mean (%)** | **SEM** | **Mean (%)** | **SEM** | **Mean (%)** | **SEM** |
| *Escherichia coli* | 4.54 | 1.81 | 2.08 | 1.44 | 20.31 | 6.15 | 7.52 | 5.11 |
| *Bacteroides vulgatus* | 2.53 | 0.72 | 4.98 | 1.33 | 2.50 | 1.17 | 5.06 | 2.07 |
| *Prevotella copri* | 1.59 | 0.73 | 5.86 | 1.90 | 1.04 | 0.78 | 1.34 | 0.76 |
| *Gemmiger formicilis* | 2.83 | 0.67 | 1.06 | 0.34 | 7.73 | 6.70 | 1.64 | 0.90 |
| *Akkermansia muciniphila* | 3.70 | 2.51 | 0.02 | 0.01 | 0.02 | 0.00 | 4.60 | 3.46 |
| *Faecalibacterium prausnitzii* | 1.53 | 0.43 | 2.08 | 0.60 | 0.47 | 0.24 | 2.18 | 1.34 |
| *Subdoligranulum* sp. APC924/74 | 1.46 | 0.42 | 1.65 | 0.72 | 1.38 | 0.77 | 1.16 | 0.47 |
| *Bacteroides ovatus* | 1.09 | 0.43 | 0.82 | 0.21 | 0.64 | 0.26 | 1.88 | 0.49 |
| *Streptococcus thermophilus* | 2.30 | 1.53 | 0.38 | 0.25 | 0.12 | 0.09 | 0.03 | 0.02 |
| *Bifidobacterium adolescentis* | 1.16 | 1.08 | 0.36 | 0.18 | 0.20 | 0.16 | 1.10 | 1.07 |
| *Actinomyces* sp. HPA0247 | 1.10 | 1.04 | 0.01 | 0.00 | 0.00 | 0.00 | 0.01 | 0.01 |

*Only taxa with abundance greater than 1% are shown.

**Supplementary Table 8** Pair comparison of intestinal microbiota composition in different cohorts

| **Taxon** | **Baseline VS Health** | | **Baseline VS CAP** | | **Health VS CAP** | | **Last follow up VS Baseline** | | **Last follow up VS Health** | | **Last follow up VS CAP** | |
| --- | --- | --- | --- | --- | --- | --- | --- | --- | --- | --- | --- | --- |
| **Phylum** | ***p* value** | **q value** | ***p* value** | **q value** | ***p* value** | **q value** | ***p* value** | **q value** | ***p* value** | **q value** | ***p* value** | **q value** |
| Bacteroidetes | 0.01 | 0.03 | 0.16 | 0.27 | 0.36 | 0.60 | 0.64 | 0.64 | 0.01 | 0.01 | 0.03 | 0.04 |
| Firmicutes | 0.96 | 0.96 | 0.60 | 0.60 | 0.51 | 0.64 | 0.15 | 0.19 | 0.15 | 0.15 | 0.18 | 0.18 |
| Proteobacteria | 0.65 | 0.81 | 0.55 | 0.60 | 0.30 | 0.60 | 0.03 | 0.07 | 0.02 | 0.02 | 0.03 | 0.04 |
| Actinobacteria | 0.01 | 0.03 | 0.04 | 0.18 | 0.80 | 0.81 | 0.02 | 0.07 | 0.00 | 0.00 | 0.00 | 0.00 |
| Verrucomicrobia | 0.07 | 0.12 | 0.09 | 0.22 | 0.27 | 0.60 | 0.05 | 0.08 | 0.00 | 0.00 | 0.00 | 0.00 |
| **Family** | ***p* value** | **q value** | ***p* value** | **q value** | ***p* value** | **q value** | ***p* value** | **q value** | ***p* value** | **q value** | ***p* value** | **q value** |
| Bacteroidaceae | 0.19 | 0.29 | 0.18 | 0.59 | 0.92 | 0.92 | 0.90 | 1.00 | 0.32 | 0.39 | 0.28 | 0.44 |
| Enterobacteriaceae | 0.08 | 0.22 | 0.65 | 0.79 | 0.50 | 0.73 | 0.02 | 0.08 | 0.00 | 0.01 | 0.14 | 0.44 |
| Ruminococcaceae | 0.76 | 0.80 | 0.65 | 0.79 | 0.60 | 0.73 | 0.52 | 0.88 | 0.21 | 0.33 | 0.57 | 0.70 |
| Prevotellaceae | 0.01 | 0.03 | 0.92 | 1.00 | 0.02 | 0.07 | 0.58 | 0.88 | 0.00 | 0.01 | 0.28 | 0.44 |
| Lachnospiraceae | 0.15 | 0.28 | 0.50 | 0.79 | 0.37 | 0.69 | 1.00 | 1.00 | 0.09 | 0.16 | 0.66 | 0.73 |
| Akkermansiaceae | 0.01 | 0.03 | 0.02 | 0.09 | 0.00 | 0.00 | 0.21 | 0.46 | 0.00 | 0.01 | 0.18 | 0.44 |
| Rikenellaceae | 0.14 | 0.28 | 0.24 | 0.59 | 0.75 | 0.83 | 0.97 | 1.00 | 0.37 | 0.40 | 0.23 | 0.44 |
| Streptococcaceae | 0.31 | 0.43 | 0.27 | 0.59 | 0.24 | 0.52 | 0.00 | 0.00 | 0.00 | 0.00 | 0.28 | 0.44 |
| Actinomycetaceae | 0.01 | 0.03 | 0.65 | 0.79 | 0.04 | 0.10 | 0.05 | 0.13 | 0.32 | 0.39 | 0.11 | 0.44 |
| Bifidobacteriaceae | 0.39 | 0.48 | 0.56 | 1.00 | 0.55 | 0.73 | 0.64 | 0.88 | 0.77 | 0.77 | 0.49 | 0.67 |
| Clostridiaceae | 0.80 | 0.80 | 0.00 | 0.04 | 0.00 | 0.01 | 0.00 | 0.01 | 0.00 | 0.00 | 0.95 | 0.95 |
| **Genus** | ***p* value** | **q value** | ***p* value** | **q value** | ***p* value** | **q value** | ***p* value** | **q value** | ***p* value** | **q value** | ***p* value** | **q value** |
| *Bacteroides* | 0.17 | 0.30 | 0.18 | 0.52 | 0.92 | 0.97 | 0.90 | 0.97 | 0.32 | 0.50 | 0.25 | 0.56 |
| *Escherichia* | 0.10 | 0.24 | 0.75 | 0.93 | 0.50 | 0.85 | 0.02 | 0.10 | 0.00 | 0.01 | 0.10 | 0.56 |
| *Prevotella* | 0.01 | 0.04 | 0.80 | 0.93 | 0.02 | 0.11 | 0.58 | 0.68 | 0.00 | 0.01 | 0.57 | 0.67 |
| *Gemmiger* | 0.06 | 0.20 | 0.14 | 0.49 | 0.65 | 0.89 | 0.52 | 0.66 | 0.77 | 0.82 | 0.32 | 0.56 |
| *Akkermansia* | 0.01 | 0.04 | 0.97 | 0.97 | 0.10 | 0.29 | 0.04 | 0.10 | 0.52 | 0.73 | 0.28 | 0.56 |
| *Alistipes* | 0.14 | 0.28 | 0.50 | 0.85 | 0.55 | 0.85 | 0.18 | 0.31 | 0.02 | 0.05 | 0.20 | 0.56 |
| *Faecalibacterium* | 0.69 | 0.74 | 0.50 | 0.85 | 0.41 | 0.83 | 0.11 | 0.21 | 0.09 | 0.17 | 0.31 | 0.56 |
| *Streptococcus* | 0.26 | 0.41 | 0.09 | 0.42 | 0.02 | 0.11 | 0.02 | 0.10 | 0.00 | 0.01 | 0.39 | 0.56 |
| *Subdoligranulum* | 0.42 | 0.59 | 0.46 | 0.85 | 0.97 | 0.97 | 1.00 | 1.00 | 1.00 | 1.00 | 0.39 | 0.56 |
| *Bifidobacterium* | 0.54 | 0.69 | 0.86 | 0.93 | 0.70 | 0.89 | 0.24 | 0.38 | 0.32 | 0.50 | 0.32 | 0.56 |
| *Actinomyces* | 0.00 | 0.04 | 0.01 | 0.14 | 0.30 | 0.70 | 0.02 | 0.10 | 0.58 | 0.74 | 0.62 | 0.68 |
| *Blautia* | 0.07 | 0.20 | 0.55 | 0.85 | 0.02 | 0.11 | 0.11 | 0.21 | 0.00 | 0.01 | 0.20 | 0.56 |
| *Veillonella* | 0.61 | 0.72 | 0.70 | 0.93 | 0.92 | 0.97 | 0.37 | 0.51 | 0.64 | 0.75 | 0.50 | 0.64 |
| *Clostridium* | 0.92 | 0.92 | 0.08 | 0.42 | 0.03 | 0.11 | 0.03 | 0.10 | 0.03 | 0.07 | 0.67 | 0.68 |
| **Species** | ***p* value** | **q value** | ***p* value** | **q value** | ***p* value** | **q value** | ***p* value** | **q value** | ***p* value** | **q value** | ***p* value** | **q value** |
| *Escherichia coli* | 0.09 | 0.20 | 0.70 | 0.77 | 0.50 | 0.69 | 0.02 | 0.12 | 0.00 | 0.02 | 0.14 | 0.62 |
| *Bacteroides vulgatus* | 0.22 | 0.38 | 0.37 | 0.69 | 0.65 | 0.71 | 0.90 | 0.90 | 0.28 | 0.51 | 0.66 | 0.81 |
| *Prevotella copri* | 0.01 | 0.05 | 0.65 | 0.77 | 0.06 | 0.23 | 0.47 | 0.72 | 0.01 | 0.04 | 0.23 | 0.62 |
| *Gemmiger formicilis* | 0.06 | 0.18 | 0.14 | 0.39 | 0.65 | 0.71 | 0.52 | 0.72 | 0.77 | 0.84 | 0.49 | 0.77 |
| *Akkermansia muciniphila* | 0.01 | 0.05 | 0.92 | 0.92 | 0.09 | 0.24 | 0.11 | 0.29 | 0.21 | 0.46 | 0.28 | 0.62 |
| *Faecalibacterium prausnitzii* | 0.69 | 0.88 | 0.50 | 0.77 | 0.46 | 0.69 | 0.09 | 0.29 | 0.09 | 0.32 | 0.49 | 0.77 |
| *Subdoligranulum* sp. APC924/74 | 0.80 | 0.88 | 0.60 | 0.77 | 0.75 | 0.75 | 0.90 | 0.90 | 0.90 | 0.90 | 0.75 | 0.83 |
| *Bacteroides ovatus* | 0.88 | 0.88 | 0.10 | 0.38 | 0.06 | 0.23 | 0.90 | 0.90 | 0.47 | 0.57 | 0.08 | 0.62 |
| *Streptococcus thermophilus* | 0.24 | 0.38 | 0.01 | 0.07 | 0.05 | 0.23 | 0.18 | 0.39 | 0.37 | 0.51 | 0.23 | 0.62 |
| *Bifidobacterium adolescentis* | 0.72 | 0.88 | 0.24 | 0.52 | 0.34 | 0.62 | 0.37 | 0.67 | 0.37 | 0.51 | 0.95 | 0.95 |
| *Actinomyces* sp. HPA0247 | 0.01 | 0.05 | 0.01 | 0.07 | 0.27 | 0.59 | 0.00 | 0.05 | 0.13 | 0.35 | 0.66 | 0.81 |

*Only taxa with abundance greater than 1% are shown.

**Supplementary Table 9** Gut microbes feature in COVID-19 and CAP patients based on LEfSe analysis

| **Patients** | **Taxon** | **Group** | **Log value** | **LDA values** | ***p* value** |
| --- | --- | --- | --- | --- | --- |
| **COVID-19** | d__Bacteria.p__Actinobacteria.c__Actinobacteria.o__Actinomycetales.f__Actinomycetaceae.g__Schaalia.s__Schaalia_odontolytica | Baseline | 3.783863896 | 3.51602357 | 0.00960474431743 |
|  | d__Bacteria.p__Actinobacteria.c__Actinobacteria.o__Actinomycetales.f__Actinomycetaceae.g__Actinomyces.s__Actinomyces_sp__ICM58 | Baseline | 3.316209013 | 3.03351508 | 0.0256711512059 |
|  | d__Bacteria.p__Actinobacteria.c__Actinobacteria.o__Actinomycetales.f__Actinomycetaceae.g__Actinomyces.s__Actinomyces_sp__HPA0247 | Baseline | 3.72190174 | 3.46410182 | 0.013818032875 |
|  | d__Bacteria.p__Firmicutes.c__Bacilli.o__Lactobacillales.f__Lactobacillaceae.g__Lactobacillus.s__Lactobacillus_rhamnosus | Baseline | 3.424186741 | 3.06874525 | 0.0226363281751 |
|  | d__Bacteria.p__Verrucomicrobia.c__Verrucomicrobiae.o__Verrucomicrobiales.f__Akkermansiaceae.g__Akkermansia.s__Akkermansia_muciniphila | Baseline | 4.563651585 | 4.2465622 | 0.0111341040457 |
|  | d__Bacteria.p__Verrucomicrobia.c__Verrucomicrobiae.o__Verrucomicrobiales.f__Akkermansiaceae.g__Akkermansia.s__Akkermansia_sp__CAG_344 | Baseline | 3.617600417 | 3.29221166 | 0.00960474431743 |
|  | d__Bacteria.p__Proteobacteria.c__Gammaproteobacteria.o__Enterobacterales.f__Enterobacteriaceae.g__Klebsiella.s__Klebsiella_pneumoniae | Last follow up | 3.556145715 | 3.03598036 | 0.023919348248 |
|  | d__Bacteria.p__Proteobacteria.c__Gammaproteobacteria.o__Enterobacterales.f__Enterobacteriaceae.g__Escherichia.s__Escherichia_coli | Last follow up | 5.304185457 | 4.92171942 | 0.00133041526659 |
|  | d__Bacteria.p__Proteobacteria.c__Gammaproteobacteria.o__Enterobacterales.f__Enterobacteriaceae.g__Shigella.s__Shigella_dysenteriae | Last follow up | 3.386106485 | 3.00213249 | 0.00296244473027 |
|  | d__Bacteria.p__Proteobacteria.c__Gammaproteobacteria.o__Enterobacterales.f__Enterobacteriaceae.g__Shigella.s__Shigella_flexneri | Last follow up | 3.657113339 | 3.26375917 | 0.00228193725332 |
| **Community acquired pneumonia** | d__Bacteria.p__Firmicutes.c__Negativicutes.o__Veillonellales.f__Veillonellaceae.g__Megasphaera |  | 3.498973683 | 3.15769751 | 0.0183222935594 |
|  | d__Bacteria.p__Bacteroidetes.c__Bacteroidia.o__Bacteroidales.f__Bacteroidaceae.g__Bacteroides.s__Bacteroides_caccae |  | 3.950429238 | 3.45186925 | 0.0357112697856 |

**Supplementary Table 10** Gut microbial composition of COVID-19 patients based on MT data

| **Phylum** | **Relative abundance (%)** | **Genus** | **Relative abundance (%)** | **Species** | **Relative abundance (%)** |
| --- | --- | --- | --- | --- | --- |
| Firmicutes | 36.41 | *Bacteroides* | 12.60 | *Prevotella copri* | 4.42 |
| Bacteroidete | 20.47 | *Prevotella* | 6.81 | *Faecalibacterium prausnitzii* | 4.59 |
| Proteobacteria | 6.01 | *Faecalibacterium* | 5.92 | *Escherichia coli* | 2.85 |
| Actinobacteria | 6.79 | *Escherichia* | 4.46 | *Gemmiger formicilis* | 4.89 |
|  |  | *Gemmiger* | 4.07 | *Subdoligranulum* sp. APC924/74 | 1.67 |
|  |  | *Subdoligranulum* | 3.41 | *Eubacterium_rectale* | 1.28 |
|  |  | *Actinomyces* | 3.33 | *Bacteroides thetaiotaomicron* | 1.46 |
|  |  | *Clostridium* | 2.36 | *Bacteroides caccae* | 1.34 |
|  |  | *Streptococcus* | 2.36 | *Actinomyces* sp. HPA0247 | 1.39 |
|  |  | *Veillonella* | 2.06 | *Bacteroides vulgatus* | 1.07 |
|  |  | *Ruminococcus* | 1.61 | *Streptococcus thermophilus* | 1.14 |
|  |  | *Blautia* | 1.30 | *Schaalia odontolytica* | 1.07 |
|  |  | *Parabacteroides* | 1.27 | *Subdoligranulum* sp. 60_17 | 1.04 |
|  |  | *Alistipes* | 1.26 |  |  |
|  |  | *Roseburia* | 1.26 |  |  |
|  |  | *Collinsella* | 1.25 |  |  |
|  |  | *Chlamydia* | 1.19 |  |  |
|  |  | *Magnetospirillum* | 1.12 |  |  |
|  |  | *Lachnoclostridium* | 1.05 |  |  |
|  |  | *Schaalia* | 1.04 |  |  |

*Only taxa with abundance greater than 1% are shown.

**Supplementary Table 11** Ratio of bacterial average relative abundance between MT and MG

| **Phylum** | **Log_2_(MT/MG ratio)** | **Genus** | **Log_2_(MT/MG ratio)** | **Species** | **Log2(MT/MG ratio)** |
| --- | --- | --- | --- | --- | --- |
| *Firmicutes* | 0.40 | *Bacteroides* | -0.23 | *Escherichia coli* | 0.18 |
| *Bacteroidetes* | -0.06 | *Escherichia* | 0.07 | *Bacteroides vulgatus* | -1.24 |
| *Proteobacteria* | -0.50 | *Prevotella* | 1.76 | *Prevotella copri* | 1.60 |
| *Actinobacteria* | -0.10 | *Gemmiger* | 0.47 | *Gemmiger formicilis* | 0.81 |
| *Verrucomicrobia* | -2.53 | *Akkermansia* | -2.72 | *Akkermansia muciniphila* | -2.53 |
| *Chlamydiae* | 3.39 | *Alistipes* | -2.01 | *Faecalibacterium prausnitzii* | 1.90 |
| *Lentisphaerae* | -1.66 | *Faecalibacterium* | 1.88 | *Subdoligranulum* sp. APC924/74 | 0.36 |
| *Candidatus Saccharibacteria* | 1.03 | *Streptococcus* | 0.20 | *Clostridium leptum* | -3.82 |
| *Spirochaetes* | 4.97 | *Subdoligranulum* | 0.75 | *Bacteroides plebeius* | -0.55 |
| *Synergistetes* | -2.97 | *Bifidobacterium* | 0.01 | *Bacteroides ovatus* | 0.13 |
| *Fusobacteria* | 2.19 | *Actinomyces* | -0.28 | *Bacteroides uniformis* | 0.32 |
| *Chlorobi* | 11.13 | *Blautia* | -0.59 | *Alistipes putredinis* | 0.53 |
| *Planctomycetes* | 1.80 | *Veillonella* | 2.91 | *Streptococcus thermophilus* | -0.26 |
| *Chloroflexi* | 0.36 | *Clostridium* | 1.14 | *Bacteroides fragilis* | 0.09 |
| *Tenericutes* | 1.74 | *Bacillus* | 0.43 | *Bacteroides stercoris* | 0.83 |
| *Candidatus Parcubacteria* | -3.26 | *Ruminococcus* | 0.83 | *Bifidobacterium adolescentis* | -0.21 |
| *Dictyoglomi* | 3.08 | *Parabacteroides* | 1.29 | *Streptococcus salivarius* | -1.58 |
| *Acidobacteria* | 0.29 | *Phascolarctobacterium* | -0.12 | *Subdoligranulum* sp. 60_17 | 0.85 |
| *Candidatus Melainabacteria* | 1.87 | *Lachnoclostridium* | 0.60 | *Bacteroides caccae* | 1.38 |
| *Cyanobacteria* | 2.70 | *Dorea* | 1.13 | *Bacteroides xylanisolvens* | -0.68 |

*Only the top 20 abundant taxon were shown.

**Supplementary Table 12** Gut bacteria (genus and species) with altered abundance in COVID-19 patients

| **Changed bacteria** | **Belonging to family** | **Belonging to phylum** | **Characteristics** | **Alternation** | | **Ref** |
| --- | --- | --- | --- | --- | --- | --- |
| *Actinomyces* | Actinomycetaceae | Actinobacteria | Opportunistic pathogen | | + | ^1^ |
| *Actinomyces* | Actinomycetaceae | Actinobacteria | Opportunistic pathogen | | + | This study |
| *Actinomyces* sp. ICM58 | Actinomycetaceae | Actinobacteria | Opportunistic pathogen | | + | This study |
| *Actinomyces* sp. HPA0247 | Actinomycetaceae | Actinobacteria | Opportunistic pathogen | | + | This study |
| *Actinomyces viscosus* | Actinomycetaceae | Actinobacteria | Opportunistic pathogen | | + | ^2^ |
| *Schaalia* | Actinomycetaceae | Actinobacteria | ND | | + | This study |
| *Schaalia odontolytica* | Actinomycetaceae | Actinobacteria | Commensal in oral | | + | This study |
| *Bifidobacterium* | Bifidobacteriaceae | Actinobacteria | Acetate producer | | + | ^3^ |
| *Eggerthella* | Eggerthellaceae | Actinobacteria | ND | | + | ^4^ |
| *Eggerthella lenta* | Eggerthellaceae | Actinobacteria | ND | | + | ^4^ |
| *Rothia** | Micrococcaceae | Actinobacteria | Opportunistic pathogen | | + | ^1^ |
| *Rothia** | Micrococcaceae | Actinobacteria | Opportunistic pathogen | | + | This study |
| *Bacteroides nordii* | Bacteroidaceae | Bacteroidetes | Opportunistic pathogen | | + | ^2^ |
| *Coprobacillus* | Coprobacillaceae | Firmicutes | ND | | + | ^4^ |
| *Clostridium* | Clostridiaceae | Firmicutes | ND | | + | ^3^ |
| *Clostridium hathewayi* | Clostridiaceae | Firmicutes | Opportunistic pathogen | | + | ^2^ |
| *Clostridium ramosum* | Clostridiaceae | Firmicutes | ND | | + | ^4^ |
| *Erysipelatoclostridium* | Clostridiaceae | Firmicutes | Opportunistic pathogen | | + | ^1^ |
| *Lactobacillus* | Lactobacillaceae | Firmicutes | Acetate producer | | + | ^3^ |
| *Lactobacillus* | Lactobacillaceae | Firmicutes | Acetate producer | | + | This study |
| *Lactobacillus rhamnosus* | Lactobacillaceae | Firmicutes | Probiotic; Acetate producer | | + | This study |
| *Lachnospiraceae bacterium* 2_1_58FAA | Lactobacillaceae | Firmicutes | Propionate producer | | + | ^4^ |
| *Lachnospiraceae bacterium* 1_4_56FAA | Lactobacillaceae | Firmicutes | Propionate producer | | + | ^4^ |
| *Streptococcus** | Streptococcaceae | Firmicutes | Opportunistic pathogen | | + | ^1^ |
| *Streptococcus** | Streptococcaceae | Firmicutes | ND | | + | ^3^ |
| *Ruminococcus gnavus* | Oscillospiraceae | Firmicutes | ND | | + | ^4^ |
| *Subdoligranulum* | Oscillospiraceae | Firmicutes | Potential probiotic | | + | This study |
| *Veillonella* | Veillonellaceae | Firmicutes | Opportunistic pathogen | | + | ^1^ |
| *Escherichia* | Enterobacteriaceae | Proteobacteria | Opportunistic pathogen | | + | This study |
| *Gemmiger* | Hyphomicrobiaceae | Proteobacteria | Commensal | | + | This study |
| *Akkermansia* | Akkermansiaceae | Verrucomicrobia | ND | | + | This study |
| *Akkermansia muciniphila* | Akkermansiaceae | Verrucomicrobia | Potential probiotic | | + | This study |
| *Akkermansia* sp. CAG-344 | Akkermansiaceae | Verrucomicrobia | ND | | + | This study |
| *Bifidobacterium* | Bifidobacteriaceae | Actinobacteria | Probiotic | | - | ^5^ |
| *Bacteroides salyersiae* | Bacteroidaceae | Bacteroidetes | ND | | - | ^4^ |
| *Bacteroides* | Bacteroidaceae | Bacteroidetes | ND | | - | ^4^ |
| *Bacteroides* | Bacteroidaceae | Bacteroidetes | ND | | - | This study |
| *Odoribacter splanchnicus* | Odoribacteraceae | Bacteroidetes | ND | | - | ^4^ |
| *Prevotella copri* | Prevotellaceae | Bacteroidetes | Acetate producer | | - | This study |
| *Prevotella* sp. BV3P1 | Prevotellaceae | Bacteroidetes | Acetate producer | | - | This study |
| *Alistipes inistinctus* | Rikenellaceae | Bacteroidetes | ND | | - | ^4^ |
| *Alistipes shahii* | Rikenellaceae | Bacteroidetes | ND | | - | ^4^ |
| *Alistipes* sp. AP11 | Rikenellaceae | Bacteroidetes | ND | | - | ^4^ |
| *Alistipes putredinis* | Rikenellaceae | Bacteroidetes | Indole producer | | - | This study |
| *Clostridium leptum* | Clostridiaceae | Firmicutes | Butyrate producer | | - | This study |
| *Eubacterium rectale* | Eubacteriaceae | Firmicutes | Butyrate producer | | - | ^2^ |
| *Eubacterium eligens* | Eubacteriaceae | Firmicutes | ND | | - | ^4^ |
| *Eubacterium hallii** | Eubacteriaceae | Firmicutes | Butyrate producer | | - | ^1^ |
| *Eubacterium hallii** | Eubacteriaceae | Firmicutes | ND | | - | ^4^ |
| *Anaerostipes* | Lachnospiraceae | Firmicutes | Butyrate producer | | - | ^1^ |
| *Agathobacter* | Lachnospiraceae | Firmicutes | ND | | - | ^1^ |
| *Coprococcus* | Lachnospiraceae | Firmicutes | ND | | - | ^3^ |
| *Coprobacter fastidiosus* | Lachnospiraceae | Firmicutes | ND | | - | ^4^ |
| *Dorea formicigenerans* | Lachnospiraceae | Firmicutes | Immune commensal | | - | ^2^ |
| *Fusicatenibacter* | Lachnospiraceae | Firmicutes | ND | | - | ^1^ |
| *Lactobacillus* | Lactobacillaceae | Firmicutes | Probiotic | | - | ^5^ |
| *Lachnospiraceae bacterium* 5_1_63FAA | Lachnospiraceae | Firmicutes | Immune commensal | | - | ^2^ |
| *Roseburia** | Lachnospiraceae | Firmicutes | Butyrate producer | | - | ^3^ |
| *Roseburia** | Lachnospiraceae | Firmicutes | Butyrate producer | | - | ^1^ |
| *Roseburia intestinalis* | Lachnospiraceae | Firmicutes | Butyrate producer | | - | ^4^ |
| *Faecalibacterium* | Oscillospiraceae | Firmicutes | ND | | - | ^3^ |
| *Faecalibacterium prausnitzii* | Oscillospiraceae | Firmicutes | Butyrate producer | | - | ^2^ |
| *Ruminococcaceae* UCG_013 | Oscillospiraceae | Firmicutes | Butyrate producer | | - | ^1^ |
| *Ruminococcus obeum* | Oscillospiraceae | Firmicutes | Butyrate producer | | - | ^2^ |
| *Ruminococcus bromii* | Oscillospiraceae | Firmicutes | Butyrate producer | | - | ^4^ |
| *Burkholderiales bacterium* 1_1_47 | Burkholderiales | Proteobacteria | ND | | - | ^4^ |
| *Parasutterella excrementihominis* | Sutterellaceae | Proteobacteria | ND | | - | ^4^ |

"*" represents the same bacterium; “+” indicates this bacterium enriched in COVID-19 group, while “–” represent reversed alternation; ND, No data.

**Supplementary Table 13** Gut bacteria (genus and species) associated with COVID-19 severity

| **Related bacteria** | **Belonging to family** | **Belonging to phylum** | **Property** | **Severity correlation** | **Abundance alternation** | **Ref** |
| --- | --- | --- | --- | --- | --- | --- |
| *Actinomyces oris* | Actinomycetaceae | Actinobacteria | Opportunistic pathogen | + | ND | This study |
| *Corynebacterium* | Orynebacteriaceae | Actinobacteria | ND | + | ND | ^4^ |
| *Corynebacterium durum* | Orynebacteriaceae | Actinobacteria | ND | + | ND | ^4^ |
| *Rothia* | Micrococcaceae | Actinobacteria | ND | + | + | ^4^ |
| *Rothia mucilaginosa* | Micrococcaceae | Actinobacteria | ND | + | ND | ^4^ |
| *Coprobacillus* | Coprobacillaceae | Firmicutes | Up-regulate ACE2 expression | + | + | ^2^ |
| *Clostridium ramosum* | Coprobacillaceae | Firmicutes | Infection and bacteremia | + | + | ^2^ |
| *Clostridium hathewayi* | Coprobacillaceae | Firmicutes | Infection and bacteremia | + | + | ^2^ |
| *Enterococcus** | Enterococcaceae | Firmicutes | Opportunistic pathogen | + | + | ^5^ |
| *Enterococcus** | Enterococcaceae | Firmicutes | ND | + | + | ^4^ |
| *Enterococcus faecium* | Enterococcaceae | Firmicutes | ND | + | ND | ^4^ |
| *Eubacterium rectale* | Eubacteriaceae | Firmicutes | ND | + | - | ^4^ |
| *Eisenbergiella tayi* | Lachnospiraceae | Firmicutes | Induced bacteremia | + | ND | This study |
| *Subdoligranulum* sp. 4_3_54A2FAA | Oscillospiraceae | Firmicutes | Associated with inflammatory bowel | + | ND | This study |
| *Streptococcus parasanguini* | Streptococcaceae | Firmicutes | Opportunistic pathogen | + | ND | This study |
| *Megasphaera* | Veillonellaceae | Firmicutes | ND | + | ND | ^4^ |
| *Burkholderiales bacterium* RIFCSPHIGHO2_12_FULL_63_20 | Burkholderiales | Proteobacteria | Opportunistic pathogen | + | ND | This study |
| *Campylobacter* | Campylobacteraceae | Proteobacteria | ND | + | ND | ^4^ |
| *Campylobacter gracilis* | Campylobacteraceae | Proteobacteria | ND | + | ND | ^4^ |
| *Escherichia coli* | Enterobacteriaceae | Proteobacteria | Opportunistic pathogen | + | + | This study |
| *Gemmiger formicilis* | Hyphomicrobiaceae | Proteobacteria | Associated with inflammatory bowel | + | + | This study |
| *Bacteroides thetaiotaomicron* | Bacteroidaceae | Bacteroidetes | Propionate producer; down-regulate ACE2 expression | - | ND | This study |
| *Bacteroides caccae* | Bacteroidaceae | Bacteroidetes | Propionate producer | - | ND | This study |
| *Bacteroides fragilis* | Bacteroidaceae | Bacteroidetes | Propionate producer | - | ND | This study |
| *Prevotella copri* | Prevotellaceae | Bacteroidetes | Hypoxanthine producer | - | - | This study |
| *Alistipes onderdonkii* | Rikenellaceae | Bacteroidetes | Maintain gut immune homeostasis | - | ND | ^2^ |
| *Clostridium butyricum* | Clostridiaceae | Firmicutes | Butyrate producer | - | - | ^5^ |
| *Clostridium leptum* | Clostridiaceae | Firmicutes | Butyrate producer | - | - | ^5^ |
| *Eubacterium* | Eubacteriaceae | Firmicutes | ND | - | ND | ^4^ |
| *Eubacterium rectale* | Eubacteriaceae | Firmicutes | Butyrate producer | - | - | ^5^ |
| *Faecalibacterium prausnitzii* | Oscillospiraceae | Firmicutes | Anti-inflammatory bacterium; butyrate producer | - | ND | ^5^ |
| *Bilophila* | Desulfovibrionaceae | Proteobacteria | ND | - | ND | ^3^ |
| *Citrobacter* | Enterobacteriaceae | Proteobacteria | ND | - | ND | ^5^ |

"*" represents the same bacterium; "+" denotes bacteria with a positive correlation of severity, while “–” represents bacteria with a negative correlation of severity; ND, No data.

**Supplementary References**

1. Gu S, Chen Y, Wu Z, et al. Alterations of the gut microbiota in patients with coronavirus disease 2019 or H1N1 influenza. *Clinical Infectious Diseases*. 2020;71(10):2669-2678.

2. Zuo T, Zhang F, Lui GCY, et al. Alterations in gut microbiota of patients with COVID-19 during time of hospitalization. *Gastroenterology*. 2020;159(3):944-955.e8.

3. Tao W, Zhang G, Wang X, et al. Analysis of the intestinal microbiota in COVID-19 patients and its correlation with the inflammatory factor IL-18. *Medicine in Microecology*. 2020;5:100023.

4. Cao J, Wang C, Zhang Y, et al. Integrated gut virome and bacteriome dynamics in COVID-19 patients. *Gut microbes*. 2021;13(1):1-21.

5. Tang L, Gu S, Gong Y, et al. Clinical significance of the correlation between changes in the major intestinal bacteria species and COVID-19 severity. *Engineering (Beijing)*. 2020;6(10):1178-1184.
